# Chemistry and Biological Activities of 1,2,4-Triazolethiones—Antiviral and Anti-Infective Drugs

**DOI:** 10.3390/molecules25133036

**Published:** 2020-07-03

**Authors:** Ashraf A. Aly, Alaa A. Hassan, Maysa M. Makhlouf, Stefan Bräse

**Affiliations:** 1 Chemistry Department, Faculty of Science, Minia University, El-Minia 61519, Egypt; alaahassan2001@mu.edu.eg (A.A.H.); maysaamahsoun@yahoo.com (M.M.M.); 2 Institute of Organic Chemistry, Karlsruhe Institute of Technology, 76131 Karlsruhe, Germany; 3 Institute of Biological and Chemical Systems (IBCS-FMS), Karlsruhe Institute of Technology, 76344 Eggenstein-Leopoldshafen, Germany

**Keywords:** 1,2,4-triazole ring, synthesis, reactions, biological activity

## Abstract

Mercapto-substituted 1,2,4-triazoles are very interesting compounds as they play an important role in chemopreventive and chemotherapeutic effects on cancer. In recent decades, literature has been enriched with sulfur- and nitrogen-containing heterocycles which are used as a basic nucleus of different heterocyclic compounds with various biological applications in medicine and also occupy a huge part of natural products. Therefore, we shed, herein, more light on the synthesis of this interesting class and its application as a biologically active moiety. They might also be suitable as antiviral and anti-infective drugs.

## 1. Introduction

**Figure 1:**
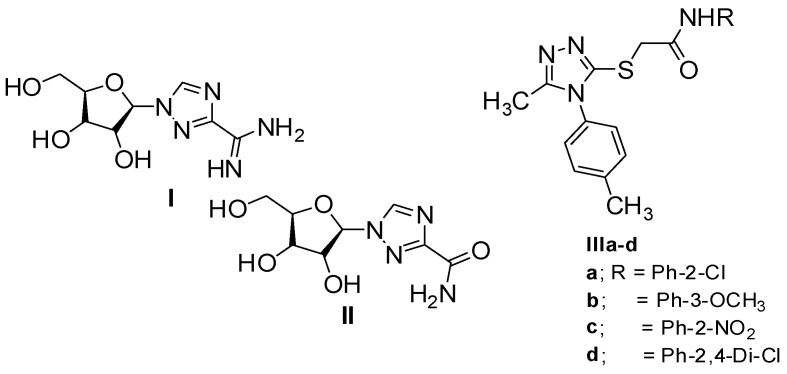
Structure of anti-HIV active triazole drugs.

Taribavirin (**I**) (Figure 1) is a triazole based clinically used as antiviral drugs (Figure 1). It is an active agent against a number of DNA and RNA viruses. It is indicated for severe respiratory syncytial virus (RSV) infection, hepatitis C infection, and other viral infections like the West Nile virus and dengue fever [1,2,3]. Taribavirin (also known as viramidine) is an antiviral drug in Phase III human trials, but not yet approved for pharmaceutical use [4].

AIDS is characterized by an abnormal host defense mechanism that predisposes to infections with opportunistic microorganisms [5]. It was reported [6] that compounds **IIIa**–**d** (Figure 1) have been proved as treatment for HIV-1. The viral enzymes, reverse transcriptase (RT), integrase (IN), and protease (PR) are all good drug targets. Two distinct types of RT inhibitors, both of which block the polymerase activity of RT, have been approved to treat HIV-1 infections, nucleoside analogs (NRTIs), and nonnucleosides (NNRTIs), and there are promising leads for compounds that either block the RNase H activity or block the polymerase in other ways. A better understanding of the structure and function(s) of RT and of the mechanism(s) of inhibition can be used to generate better drugs; in particular, drugs that are effective against the current drug-resistant strains of HIV-1.NNRTIs via high throughput screening (HTS) using a cell-based assay for inhibiting HIV-1 replication and promising activities against selected NNRTI-resistant mutants such as Y181L, Y181C, K103N, and L100I were observed.

Sulfanyltriazoles **IIIa** and **IIIc** (Figure 1) exhibited EC50 values of 182 and 24 nM, respectively, suggesting the potential of these sulfanyltriazoles to overcome the K103-related NNRTI-resistant mutants. These sulfanyltriazoles could serve as advanced lead structures promising great potential in overcoming these and other NNRTI-resistant mutants [7].

**Figure 2:**
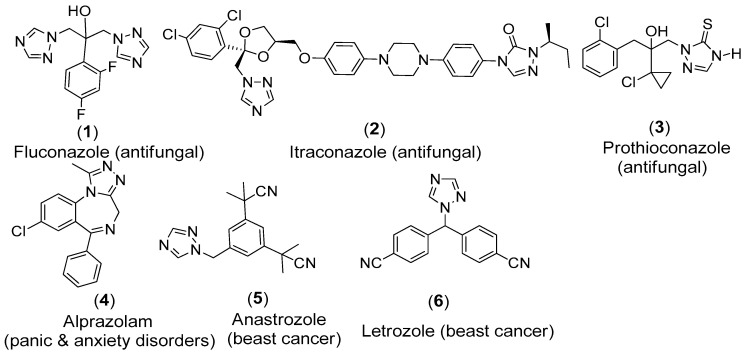
Some potent drugs containing triazole and triazolethione moieties.

1,2,4-triazoles are a very important class of compounds which attracted the attention of many chemists and biologists in organic synthesis and medicinal and pharmaceutical fields due to their various biological activities such as anticancer [8,9], antimicrobial, anticonvulsant [10], anti-inflammatory [11], antitubercular [12], analgesic [13], antibacterial [14], and anti-HIV [15]. In addition, there are chemotherapeutically known drugs containing 1,2,4-triazole moiety, e.g., fluconazole (**1**) [16], (2-(2,4-difluorophenyl)-1,3-di(1*H*-1,2,4-triazol-1-yl)propan-2-ol) and itraconazole (**2**) [17], (4-(4-(4-(4-(((2*S*,4*R*)-2-((1*H*-1,2,4-triazol-1-yl)methyl)-2-(2,4-dichlorophenyl)-1,3-dioxolan-4-yl)methoxy)phenyl)piperazin-1-yl)-phenyl)-1-((*S*)-sec-butyl)-1*H*-1,2,4-triazol-5(4*H*)-one), which are used as very effective antifungal drugs. In addition, prothioconazole (**3**) [18] is commercially available for the treatment of plant-pathogenic fungal infections, alprazolam (**4**) [18], (8-chloro-1-methyl-6-phenyl-4*H*-benzo[*f*][1,2,4]triazolo[4,3-*a*][1,4]diazepine), is used for treating of anxiety disorders, and anastrozole (**5**) [19] in addition to letrozole (**6**) is used for chemotherapeutic anticancer drugs [20] (Figure 2).

**Figure 3:**
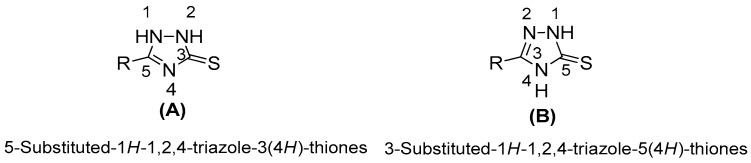
1,2,4-Triazole thiones.

Incorporating of thione group either in 3- or 5-position (A and B, Figure 3) has been reported in numerous reports, leading to enhancement of biological activities related to triazole moiety [21]. Besides, the triazolethione system is considered as a cyclic analog of very important components like thiosemicarbazides and thiocarbohydrazides, which are widely spread as a reactive building block in many organic reactions leading to different heterocyclic rings and having effective biological applications. Many heterocyclic compounds are the main constituents of natural products; also, mercapto-1,2,4-triazole nucleus is found in many natural products and pharmaceuticals [22]. Mercapto-1,2,4-triazole also may be derived from natural products by applying some reactions to get the desired compounds [23].

**Figure 4:**
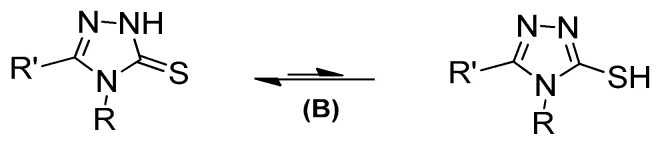
Thione-thiol tautomeric forms.

Triazolethione-thiols (Figure 4) have gained considerable importance in medicinal chemistry due to their potential anticancer [24,25], antimicrobial [26], antioxidant, antitumor [27], anti-tuberculosis [28], anticonvulsant [29], fungicidal [30], antiepileptic [31] and anti-inflammatory [32] activities.

1,2,4-triazolethiones have been prepared successfully by various methods. The most common classical method is the dehydrative cyclization of different hydrazinecarbothioamides in presence of basic media using various reagents such as sodium hydroxide [2,25,33], potassium hydroxide [34,35], sodium bicarbonate [36] or acidic ionic liquid [37] followed by neutralization either with acid or base for both cases, in addition to different other techniques including donor–acceptor interactions. Various synthetic routes and biological applications of spiro-1,2,4-triazolethiones were also discussed as main heterocyclic targets easily obtained from different hydrazinecarbothioamides [38,39]. 

Schiff bases of triazolethiones [40] have played vital roles in organic synthesis and they are obtained from triazolethiones using simple procedures; also, sometimes their structures possess biological and pharmaceutical activities other than triazolethione itself such as anti-inflammatory and anti-oxidant [41], anticancer [42], fungicidal activities [43], antibacterial [44], antiparasitic [45], antidepressant and antimicrobial [46]. Furthermore, many transition metal complexes of 1,2,4-triazolethione Schiff bases and their bioactivities were reported [47,48,49] along with nickel complexes of triazolethiones [50] showing high catalytic activity towards the synthesis of tetrahydrobenzo[*b*]pyrans [47].

Various reactions of mercapto-triazolethiones [25] were discussed depending on *S* and *N* nucleophilic sites and in presence of different reagents and conditions to afford other heterocyclic compounds, e.g., triazolothiadiazines [51,52], imidazothiadiazoles [51,53], bistriazolethione-1,4-dihydropyridines [54] and fused triazolethione pyrimidines [55]. 

## 2. Synthesis of 1,2,4-Triazole-3-thiones

**Scheme 1:**
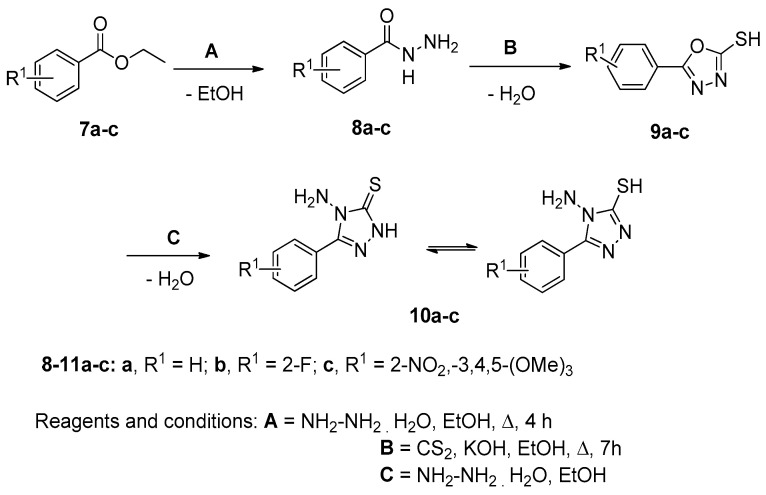
Synthesis of triazolethiones **10a**–**c**.

Hydrazinolysis of ethyl-substituted benzoates **7a**–**c** yielded the carbonylhydrazides **8a**–**c**. Nucleophilic addition of carbon disulfide (CS_2_) to **8a**–**c** in basic media [22] gave the hydrazide oxadiazole-2-thiones **9a**–**c**. The reaction of oxadiazoles **9a**–**c** with hydrazine hydrate in ethanol afforded 4-amino-5-aryl-1,2,4-triazole-3-thiones **10a**–**c** (Scheme 1) [22].

**Scheme 2:**
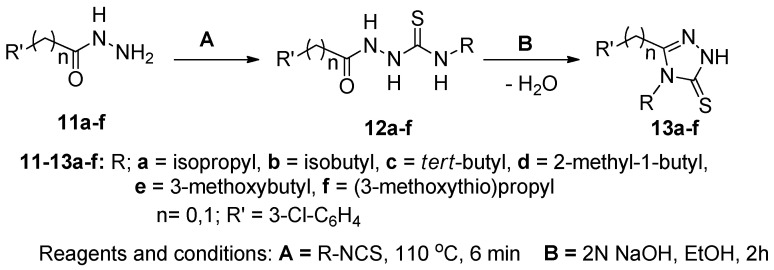
Synthesis of triazolethiones **13a**–**f**.

Alkaline cyclization of different substituted acylthiocarbohydrazides **12a**–**f**, obtained from the reactions of acylhydrazides 11a–f with various isothiocyanates, gave the corresponding 4-alkyl-5-substituted-1,2,4-triazole-3-thiones **13a**–**f** in 70–86% yields (Scheme 2) [23]. Screening of the anticonvulsant activity of the obtained compounds revealed that they are used as useful anticonvulsant drug candidates whose mode of action depends on voltage-gated sodium channels inhibition (VGSC) (Scheme 2) [23].

**Scheme 3:**
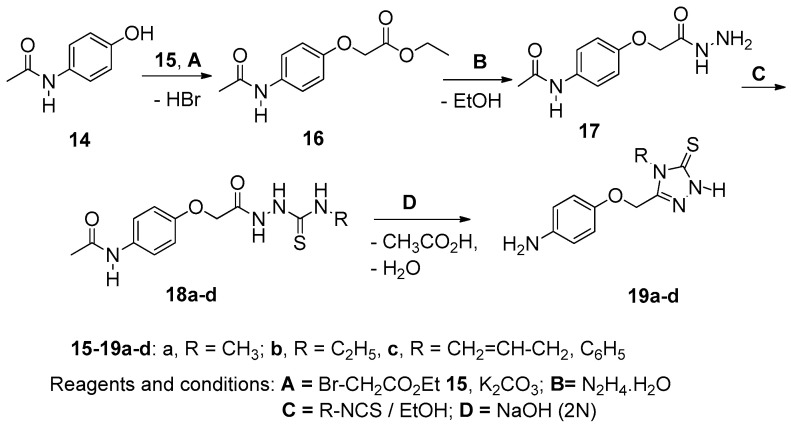
Synthesis of triazolethiones **19a**–**d**.

A series of 1,2,4-triazole-3-thiones **19a**–**d** were successfully prepared through stepwise reaction starting from esterification of *N*-(4-hydroxyphenyl)acetamide (**14**) with ethyl bromoacetate (**15**) to give ethyl 2-(4-acetamido-phenoxy)acetate (**16**) [24]. The acetohydrazide **17** was then obtained through hydrazinolysis of compound **16** with hydrazine hydrate. The corresponding thiosemicarbazides **18a**–**d** were synthesized by the reaction of **17** with different isothiocyanates in dry ethanol. Finally, thiosemicarbazide derivatives **18a**–**d** were efficiently cyclized in basic media to give the desired 1,2,4-triazole-3-thiones **19a**–**d** in 52–88% yields [24] (Scheme 3).

**Scheme 4:**
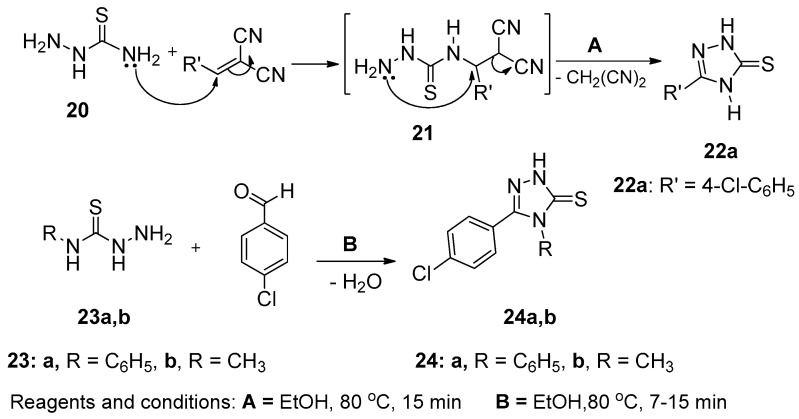
Synthesis of triazolethiones **24a**,**b**.

Reactions of thiosemicarbazide (**20**) with arylidene malononitrile afforded 5-(4-chlorophenyl)-1,2,4-triazolidine-3-thione **22a** *via* the intermediate **21**, whereas the reaction of 4-substituted thiosemicarbazides **23a**,**b** with 4-chlorobenzaldehyde gave the corresponding 5-(4-chlorophenyl)-4-substituted-1,2,4-triazolidine-3-thiones **24a**,**b** in 89% and 91% yield, respectively (Scheme 4) [50].

**Scheme 5:**
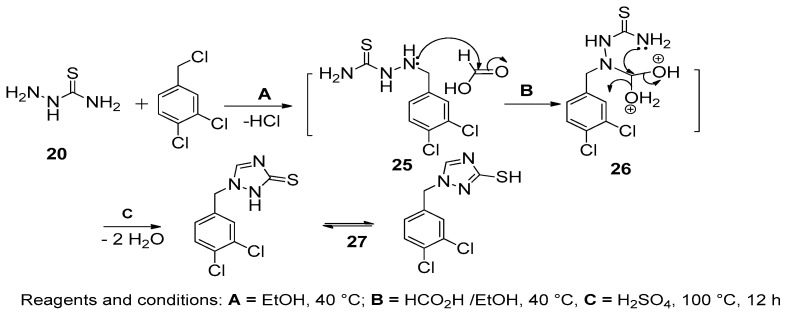
Synthesis of triazolethiones **27**.

Thiosemicarbazide (**20**) reacted with 3,4-dichlorobenzyl chloride to give 2-(3,4-dichlorobenzyl)hydrazinecarbothioamide (**25**) which reacted with formic acid to form 1-(3,4-dichlorobenzyl)-1*H*-1,2,4-triazole-3-thiol (**27**) in 82% yield through the formation of intermediate **26** (Scheme 5) [51].

**Scheme 6:**
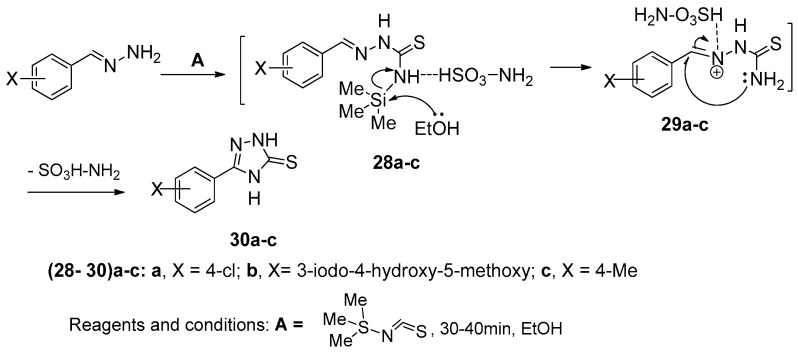
Synthesis of triazolethiones **30a**–**c**.

The synthesis of 5-substituted phenyl-1,2,4-triazole-3-thiones **30a**–**c** was done in high yields from the refluxing of arylidene derivatives and trimethylsilyl isothiocyanate using sulfamic acid as a catalyst *via* the intermediates **28a**–**c** and **29a**–**c** (Scheme 6) [52]. 

**Scheme 7:**
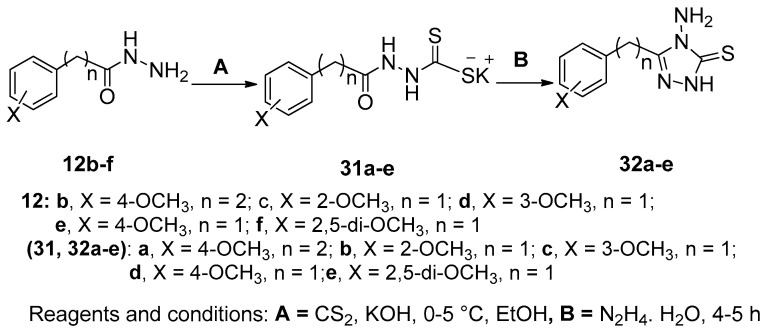
Synthesis of triazolethiones **32a**–**c**.

Substituted aryl hydrazides **12b**–**f** reacted with CS_2_ in alcoholic potassium hydroxides and yielded potassium hydrazinecarbodithioate salts **31b**–**f**. Refluxing of **31a**–**e** with a dilute solution of hydrazine hydrate afforded the expected 4-amino-5-substituted-1,2,4-triazole-3-thiones **32a**–**e** (Scheme 7) [29].

**Scheme 8:**
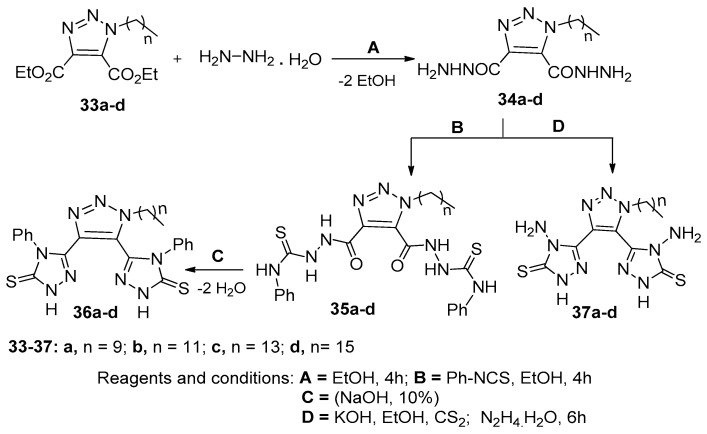
Synthesis of bis-4-amino-triazolethiones **35a**–**d**, **36a**–**d** and **37a**–**d**.

The reaction of diethyl 1-substituted-1*H*-1,2,3-triazole-4,5-dicarboxylates **33a**–**d** with hydrazine hydrate yielded diacid hydrazides **34a**–**d**. Hydrazinecarbothioamides **35a**–**d** were obtained *via* refluxing of **34a**–**d** with phenyl isothiocyanate. Dehydrative ring closure of compounds **35a**–**d** under basic condition furnished the formation of bis-1,2,4-triazole-3-thiones **36a**–**d** in 80–85% yields. Besides, the reaction of diacid hydrazides **34a**–**d** with CS_2_ in basic solution followed by refluxing with hydrazine hydrate gave bis-4-amino-1,2,4-triazole-3-thiones **37a**–**d** in 80–85% yields (Scheme 8) [28]. The resulting compounds were screened for their antimicrobial activities based on standard antimicrobial agents; compound **37d** exhibited comparable antibacterial and antifungal activities against all the tested organisms [28].

**Scheme 9:**
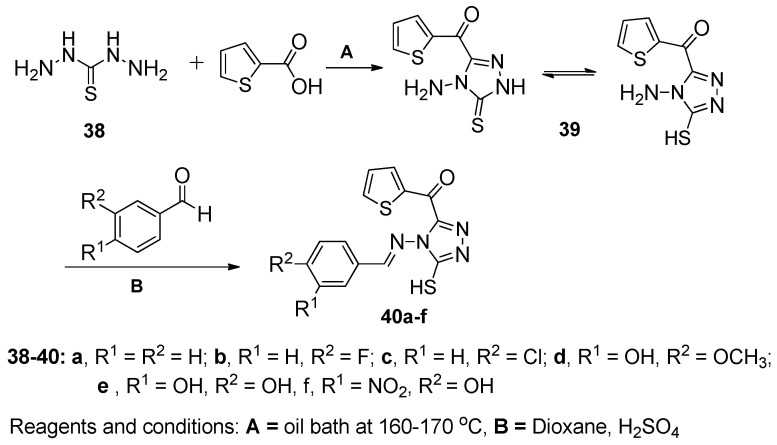
Synthesis of triazolethiones **40a**–**f**.

Thiocarbohydrazide (**38**) was heated with 2-(thiophen-2-yl)acetic acid to get 4-amino-1,2,4-triazole-3-thione (**39**). The reaction of **39** with different aryl aldehydes yielded the corresponding Schiff bases **40a**–**f** in 52–61% yields (Scheme 9) [53]. All the synthesized compounds were screened against *Mycobacterium tuberculosis* H37Rv, and they proved to be less active than rifampicin (98%), used as reference drug. Compound **40f** showed the highest inhibition (87%), and therefore, it was suggested to be as potentially active antituberculosis agent [53]. 

**Scheme 10:**
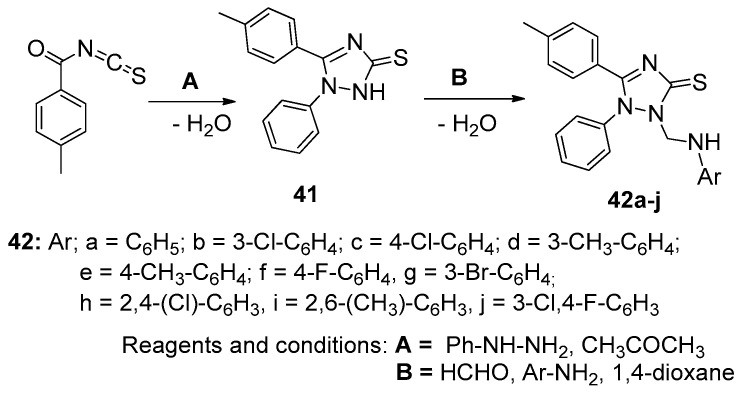
Synthesis of triazolethiones **42a**–**j**.

4-Methyl benzoylisothiocyanate was reacted with phenylhydrazine hydrate afforded 1-phenyl-5-(*p*-tolyl)-1*H*-1,2,4-triazole-3(2*H*)-thione (**41**). Schiff bases of triazolethione **42a**–**j** were obtained *via* reaction of triazolethione **41** with formaldehyde and various aromatic amines (Scheme 10) [54]. Screening of the synthesized compounds **42a**–**j** against different microorganisms showed that they have good antifungal activity rather than antibacterial activity [56].

**Scheme 11:**
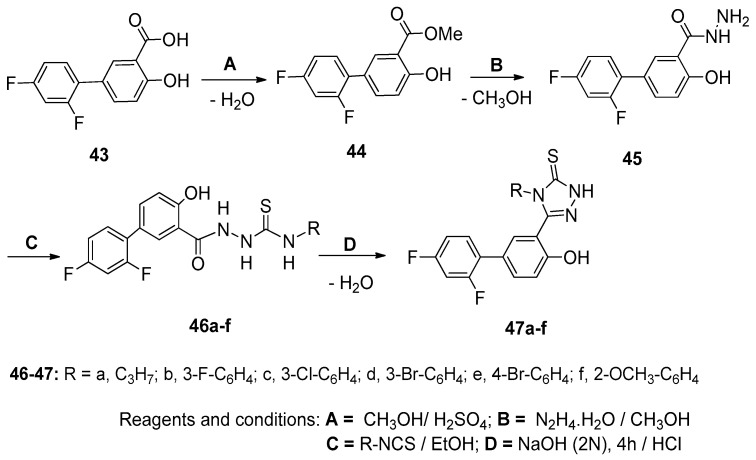
Synthesis of triazolethiones **47a**–**f**.

Diflunisal (2′,4′-difluoro-4-hydroxybiphenyl-3-carboxylic acid) (**43**) was converted to its corresponding diflunisal ester **44**. The desired diflunisal hydrazide **45** was obtained using hydrazine hydrate. The reaction of hydrazide **45** with various aryl isothiocyanates afforded substituted hydrazinecarbothioamides **46a**–**f**. Cyclization of compounds **46a**–**f** to the corresponding triazole-3-thiones **47a**–**f** occurred in basic media (Scheme 11) [57]. The screening of compounds **47a**–**f** against cancer cells revealed that compound **47f** was found to be active against the colon carcinoma HCT-116 cancer cell line with a 6.2 μM IC_50_ value. In addition, compounds **47e** and **47f** were found active against the human breast cancer T47D cancer cell line with IC_50_ values of 43.4 and 27.3 μM, respectively [57].

**Scheme 12:**
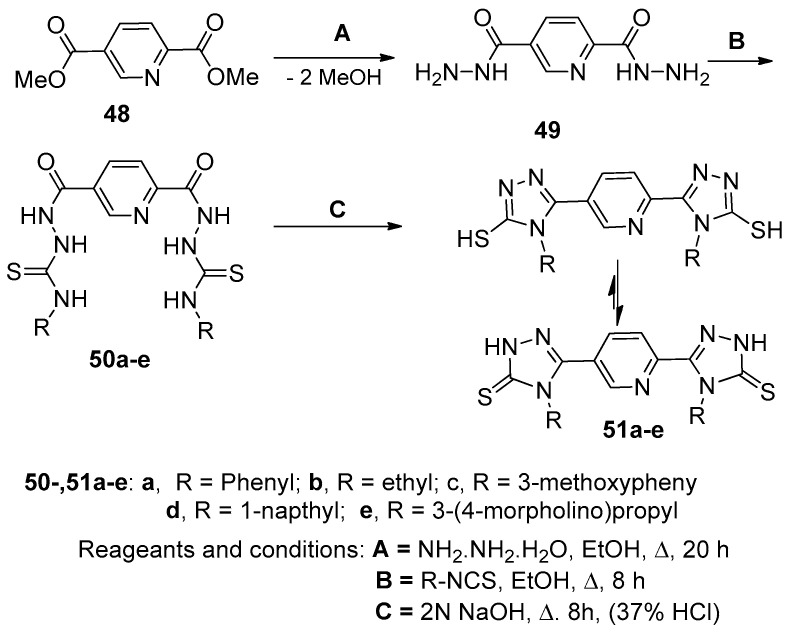
Synthesis of bis-triazolethiones **51a**–**e**.

Pyridine-2,5-dicarbohydrazide (**49**) was synthesized from the reaction of dimethylpyridine-2,5-dicarboxylic acid (**48**) with hydrazine hydrate, which reacted with different alkyl/aryl isothiocyanates to afford 2,2′-(pyridine-2,5-dicarbonyl)bis-(*N*-substituted hydrazinecarbothioamides) **50a**–**e**. Ring closure of these hydrazine-carbothioamides **50a**–**e** occurred in basic media to give bis-1,2,4-triazole-3-thiones **51a**–**e** in 85–95% yields (Scheme 12) [58]. Biological activities of the synthesized compounds **51a**–**e** were evaluated, and they showed high antioxidant activity. Moreover, all of the synthesized compounds efficiently inhibited some metabolic enzymes such as AChE (acetylcholinesterase I and II) and could be used as excellent candidate drugs in the treatment of some diseases such as mountain sickness, glaucoma, gastric and duodenal ulcers, epilepsy, osteoporosis, and neurological disorders [58]. 

**Scheme 13:**
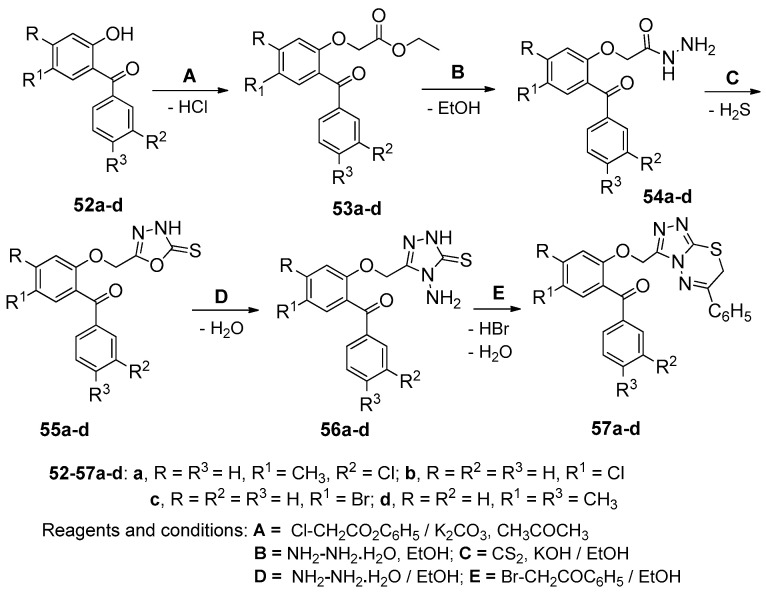
Synthesis of triazolethiones **57a**–**d**.

Ethyl (2-aroylaryloxy)acetates **53a**–**d** were synthesized from hydroxyl-benzophenones **52a**–**d** with ethyl chloroacetate. The reaction of **53a**–**d** with hydrazine hydrate gave the corresponding acylhydrazides **54a**–**d**. Intramolecular cyclization of **54a**–**d** with CS_2_ in alkaline media resulted in oxadiazole-2-(3*H*)thiones **55a**–**d**. 1,2,4-Triazolo-3-thiones **56a**–**d** were synthesized from the reaction of compounds **55a**–**d** with hydrazine hydrate. In addition, triazolothiadiazines **57a**–**d** were synthesized from the reaction of **56a**–**d** with phenacyl bromide [50]. The screening of compounds **55a**–**d** and **57a**–**d** revealed that they possess a higher antibacterial activity than antifungal activity; also, the halo-substituted compounds showed an increased growth inhibition activity higher than that of the reference drugs such as fluconazole and chloramphenicol (Scheme 13) [50].

**Scheme 14:**
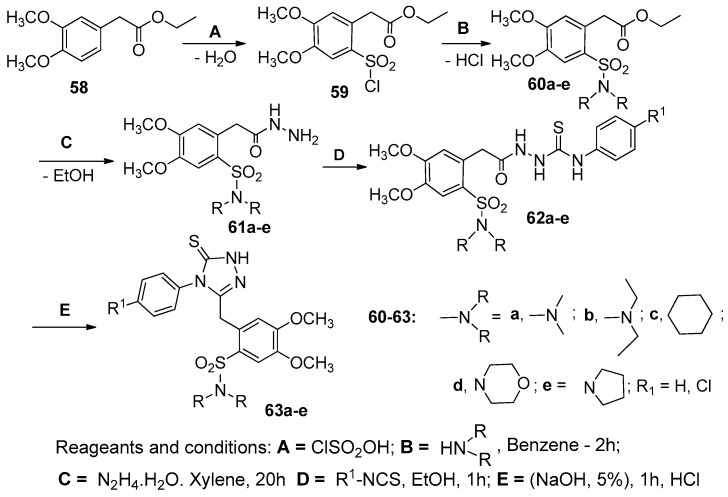
Synthesis of 1,2,4-triazole-3-thiones **63a**–**e**.

Chlorosulfonation of ethyl 2-(3,4-dimethoxyphenyl)acetate (**58**) gave ethyl 2-(2-(chlorosulfonyl)-4,5-dimethoxyphenyl)acetate (**59**) (Scheme 14). Sulfonamides **60a**–**e** were readily obtained *via* reaction of **59** with secondary amines. The desired acid hydrazides **61a**–**e**, which were obtained by reaction of **60a**–**e** with hydrazine hydrate, were condensed with various isothiocyanates to yield the corresponding hydrazinecarbothioamides **62a**–**e**. Further, 1,2,4-Triazole-3-thiones **63a**–**e** were synthesized in 44–75% yields from the cyclization **62a**–**e** in basic media (Scheme 14) [47]. Screening of compounds **63a**–**e** for *in vitro* antifungal and antibacterial activity revealed that they have the best antifungal activity compared with the reference bifonazole in addition to the same bactericidal activity as *streptomycin*, except for *Enterobacter cloacae* and *Salmonella* species [47].

**Scheme 15:**
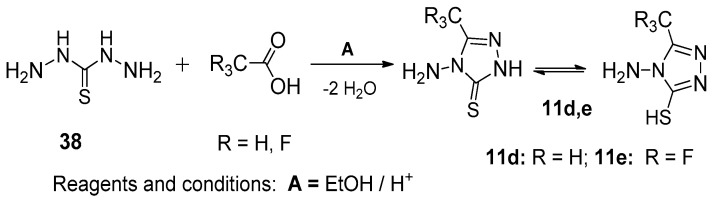
Synthesis of 4-*N*-amino-triazolethiones **11d**,**e**.

Refluxing of thiocarbohydrazide (**38**) with acetic acid or trifluoroacetic acid gave 4-amino-5-substituted-4*H*-1,2,4-triazole-3-thiones **11d**,**e** (Scheme 15) [42]. 

**Scheme 16:**
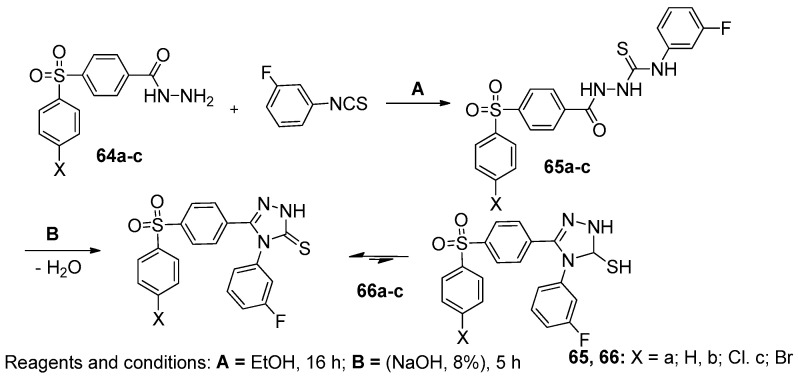
Synthesis of triazolethiones **66a**–**c**.

Various thiosemicarbazide derivatives **65a**–**c** were then synthesized from the reaction of acid hydrazides **64a**–**c** with 3-fluorophenyl isothiocyanates. Further, 1,2,4-triazole-3-thiones **66a**–**c** were obtained from alkaline cyclization of compounds **65a**–**c** with 8% NaOH solution (Scheme 16) [40].

**Scheme 17:**
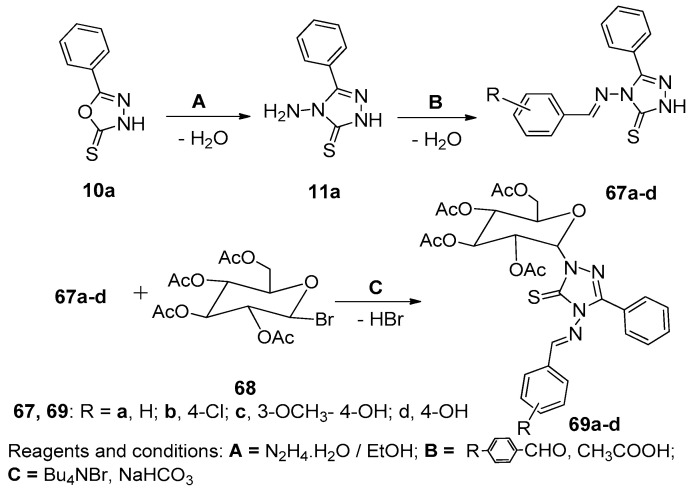
Synthesis of glycosides of triazolethiones **69a**–**d**.

Treatment of oxadiazole thione **10a** with hydrazine hydrate gave 4-amino-triazolethione (**11a**) which on reacting with various aldehydes gave the Schiff bases of triazolethiones **67a**–**d**. The synthesis of 1,3,4-trisubstituted triazolethiones **69a**–**d** was carried out from the reaction of triazolethiones **67a**–**d** with (2-(acetoxymethyl)-6-bromotetrahydro-2*H*-pyran-3,4,5-triyl triacetate (**68**) in good yields (Scheme 17) [30]. The synthesized compounds were screened for their cytotoxic activity against human malignant cell lines (MCF-7 and Bel-7402). Interestingly, **69c** showed more potent cytotoxic activity against MCF-7 cells compared with compound **67c**. Compound **69b** also was more active than compound **67b** against MCF-7 and Bel-7402 cells [30].

**Scheme 18:**
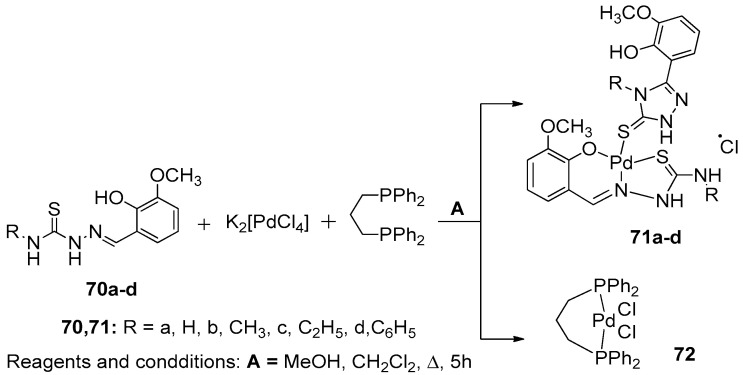
Synthesis of Pd complexes of triazolethiones **71a**–**d**.

A series of Pd complexes containing 1,2,4-triazole-3-thiones **71a**–**d** [59] were synthesized from the reaction of different thiosemicarbazones **70a**–**d** with diphenylphosphinopropane and K_2_[PdCl_4_] (Scheme 18). The reaction produced, as a minor product, compound **72** (Scheme 18) [59]. The *in vitro* cytotoxicity of **71a**–**d** was evaluated against the MCF-7 cell line, with cisplatin as a reference. The complexes **71b** and **71c** showed significant cytotoxicity against the MCF-7 (human breast cancer) cell line compared with cisplatin [59].

**Scheme 19:**
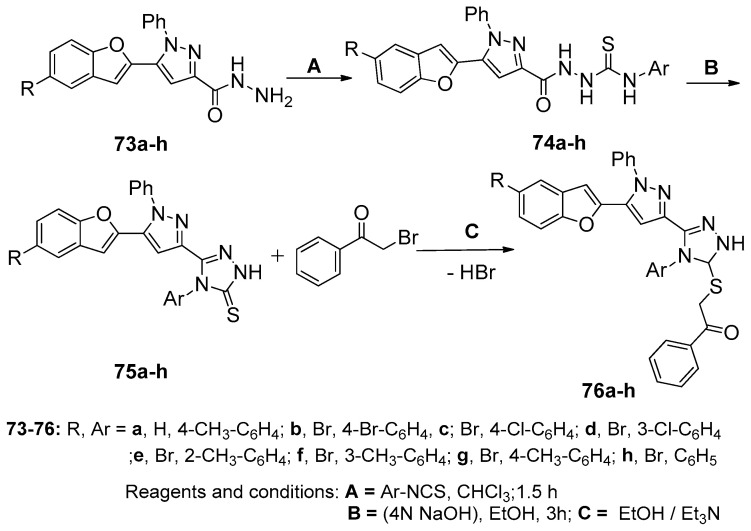
Synthesis of *S*-alkylated triazolethiones **76a**–**h**.

Refluxing of 5-benzofuran-2-yl-1-phenyl-1*H*-pyrazole-3-carbohydrazides **73a**–**h** with different aromatic isothiocyanates afforded the desired hydrazinecarbothioamides **74a**–**h**. Ring closure of these compounds **74a**–**h** occurred through refluxing with aqueous sodium hydroxide to give the target 1,2,4-triazole-3-thiones **75a**–**h** in 78–88% yields. On the other side, when 2-bromoacetophenone was reacted with **75a**–**h**, the reaction gave the corresponding benzothioates **76a**–**h** in 74–86% yields (Scheme 19) [36]. Biological activity of compounds **74a**–**h** and **76a**–**h** showed that benzothioate **76a** has a good antibacterial activity against all pathogenic bacteria compared with the standard chloramphenicol [36].

**Scheme 20:**
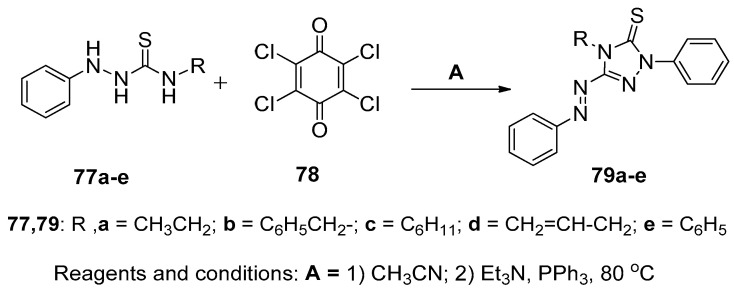
Synthesis of triazolethiones **79a**–**e**.

It was reported that the Eschenmoser coupling reaction was used as an efficient method to get 82–88% of diazenyl-1,2,4-triazole-5-thiones **79a**–**e** *via* nucleophilic attack of disubstituted hydrazinecarbothioamides **77a**–**e** on 2,3,5,6-tetrachloro-1,4-benzoquinone (**78**, *p*-CHL) which acted as a mediator [33] (Scheme 20).

**Scheme 21:**
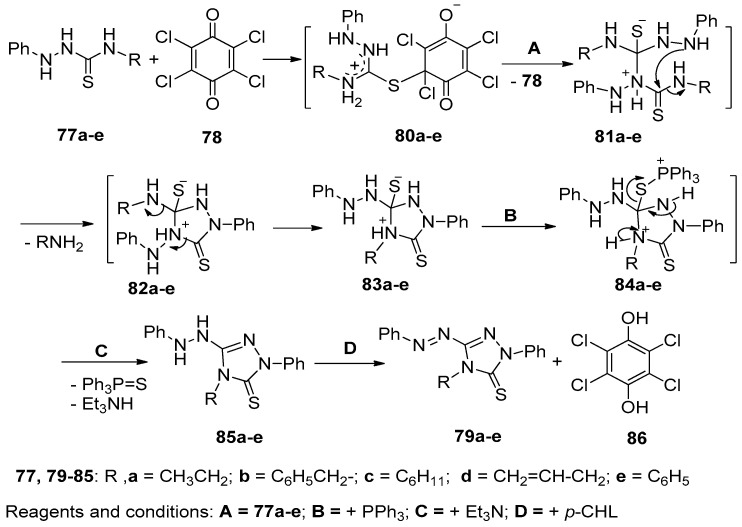
Mechanism describes formation of triazolethiones **79a**–**e**.

The suggested mechanism based on initial CT-complexation formation of **80a**–**e**, which losses chlorine molecule accompanies with the addition of another molecule of **77** would give the intermediate **81** (Scheme 21). Elimination of an arylamine equivalent from **81** would give the intermediate **82**, which undergoes rearrangement to give **83**. The addition of a Ph_3_P molecule to **83** would give **84**. The action of Ph_3_P and Et_3_N is to initiate the formation of triazolethione **85** via elimination of Ph_3_P=S and triethylammonium. Dehydrogenation of **85** by a second molecule of **78** would give the expected final product **79** together with dichlorodihydroxybenzene (**86**) [33] (Scheme 21).

**Scheme 22:**
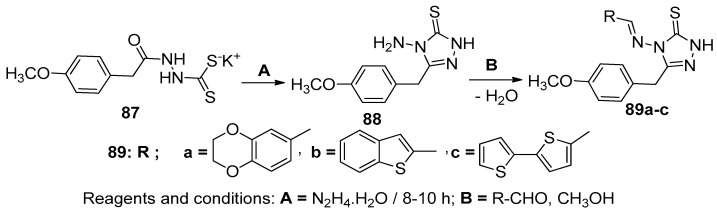
Synthesis of triazolethiones **89a**–**c**.

**Scheme 23:**
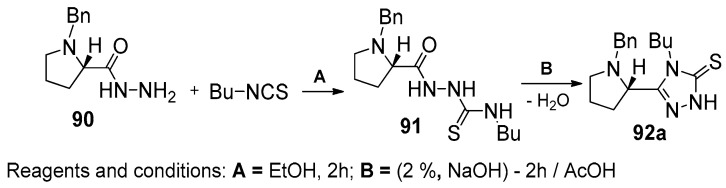
Synthesis of triazolethione **92a**.

4-Amino-3-(4-methoxybenzyl)-1*H*-1,2,4-triazole-5(4*H*)-thione (**88**) was synthesized in 75% yield by refluxing of potassium 2-(2-(4-methoxyphenyl)acetyl)-hydrazinecarbodithioate (**87**) with hydrazine hydrate. Condensation of triazolethione **88** with different substituted aldehydes gave Schiff base derivatives **89a**–**c** in 85–86% yields (Scheme 22) [43]. Screening of different Schiff bases **89a**–**c** for anti-inflammatory and antioxidant activities showed that **89a** and **89c** were used as potent anti-inflammatory drugs. In addition, compound **89a** was the most active antioxidant drug showing an IC_50_ value of 7.2 ± 2.7 μg/mL compared with that of the reference ascorbic acid (2.61 ± 0.29 g/mL) [43].

(*S*)-2-(1-Benzylpyrrolidine-2-carbonyl)-*N*-butylhydrazinecarbothioamide (**91**) was prepared from the refluxing of (*S*)-1-benzylpyrrolidine-2-carbohydrazide (**90**) with butyl isothiocyanate. In addition, it is used as a building block for heterocyclization and synthesis of the desired (*S*)-3-(1-benzylpyrrolidin-2-yl)-4-butyl-1*H*-1,2,4-triazole-5(4*H*)-thione (**92a**) in 56% yield (Scheme 23) [36]. 

**Scheme 24:**
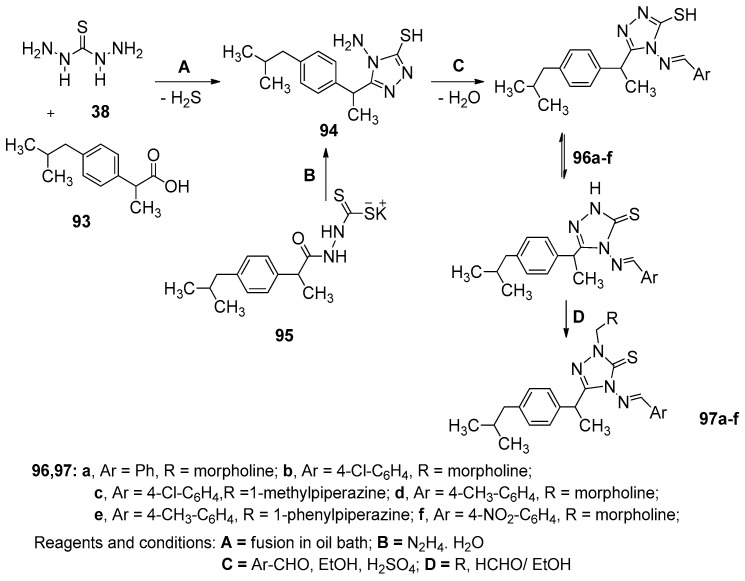
Synthesis of triazolethiones **97a**–**f**.

Moreover, 4-amino-triazole-5-thiol **94** was obtained from two routes, i.e., from the fusion of substituted propanoic acid (**93**) with **38** or cyclization of potassium hydrazinecarbodithioate derivative **95** with hydrazine hydrate (Scheme 24) [60]. 

The reaction of triazolethione **94** with different aldehydes in acidic media afforded (*E*)-4-(substituted amino)-3-(1-(4-isobutylphenyl)ethyl)-4*H*-1,2,4-triazole-5-thiones **96a**–**f** in 44–85% yields [61]. One-pot multicomponent reaction of **96a**–**f**, formaldehyde and secondary amines afforded 2,4,5-trisubstituted triazolethiones **97a**–**f**. Screening of the anti-inflammatory activity of the synthesized compounds revealed that Mannich bases (**97b** and **97e**) exhibited the highest anti-inflammatory activity. Besides, the most potent anti-inflammatory molecules **97b**,**d**–**f** were further examined for their analgesic activity in mice showing better analgesic activity compared to diclofenac [61].

**Scheme 25:**
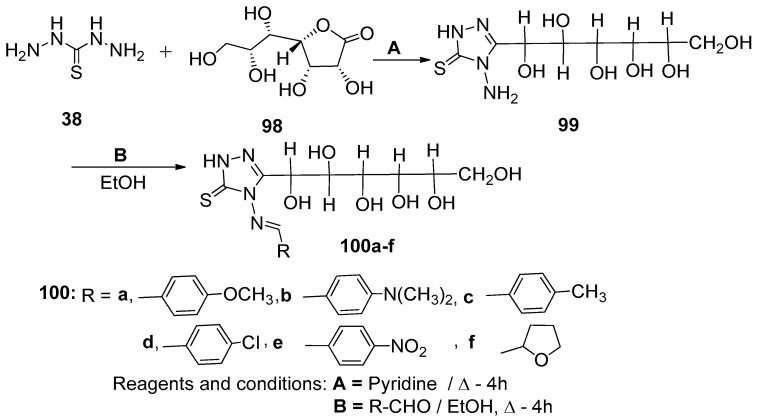
Synthesis of triazolethiones **100a**–**f**.

Thiocarbohyrazide (**38**) was used efficiently as precursor of 4-amino-3-(1,2,3,4,5,6-hexahydroxyhexyl)-1*H*-1,2,4-triazole-5(4*H*)-thione (**99**) through refluxing with *D*-glucoheptonic acid-1,4-lactone (**98**) [62]. Besides, the triazole-thione **99** was reacted with different substituted benzaldehydes to afford (*E*)-4-amino-3-(1,2,3,4,5,6-hexahydroxyhexyl)-1*H*-1,2,4-triazole-5(4*H*)-thiones **100a**–**f** in good to moderate yields (50–70%). Introducing a glycosyl unit into triazolethiones Schiff bases **100a**–**f** led to good water-solubility of these compounds and also improved their biological activities (Scheme 25) [62].

**Scheme 26:**
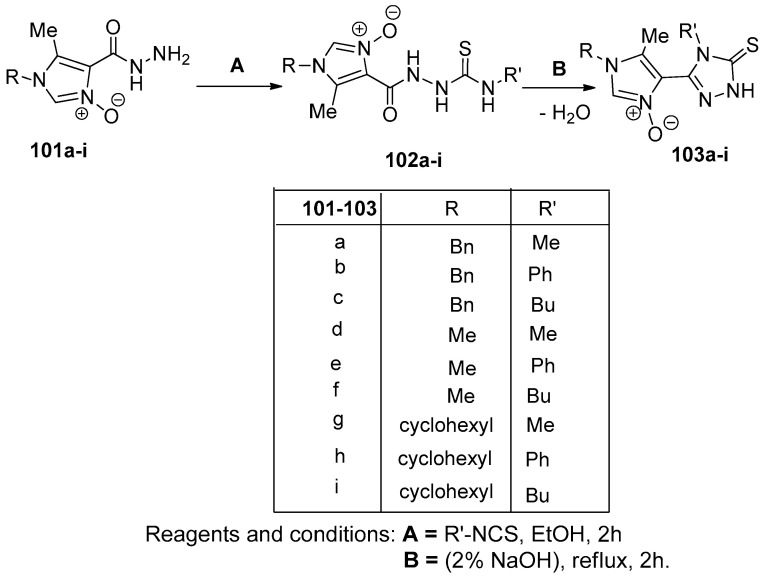
Synthesis of imidazolyl-*N*-oxide-triazolethiones **103a**–**i**.

4-(Hydrazinylcarbonyl)-5-methyl-4,5-dihydro-1*H*-imidazole-3-oxides **101a**–**i** reacted with different isothiocyanates (phenyl, *t*-butyl, and methyl) to give the corresponding hydrazinecarbothioamides **102a**–**i**. Triazole-5-thiones **103a**–**i** were obtained in 50–82% yields from the cyclization of substituted imidazole (carbonyl)hydrazinecarbothioamides **102a**–**i** in basic media (Scheme 26) [63]. 

**Scheme 27:**
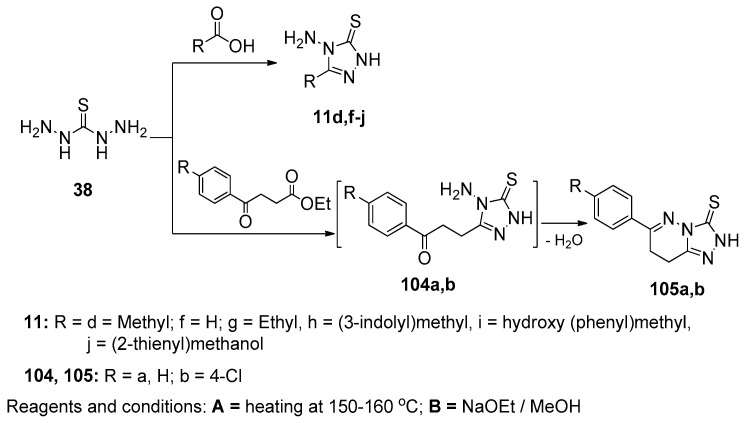
Synthesis of pyridazino-triazolethiones **105a**,**b**.

The reaction of different carboxylic acids with thiocarbohydrazide **38** gave aminotriazolethiones **11d**,**f**–**j** in 51–57% yields. In a different manner, the reaction of **38** with ethyl esters of γ-keto acids did not give the expected triazolethiones **104a**,**b** but it gave 6-substituted phenyl-7,8-dihydro-[1,2,4]triazolo[4,3-*b*]pyridazine-3(2*H*)-thiones **105a**,**b** in 35% and 39% yields. The reaction occurred *via* ring closure of triazole and intramolecular imine condensation of **104a**,**b** (Scheme 27) [64]. The prepared compounds were tested for their inhibitory activities against *Mycobacterium bovis BCG*; compound **11d** proved to be the most potent one against it, with MIC value of 31.25 μg/mL [64].

**Scheme 28:**
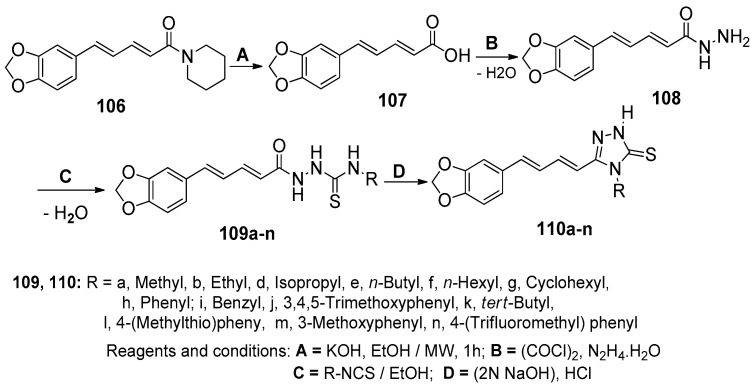
Synthesis of triazolethiones **110a**–**n**.

Natural products are used as staring materials for the synthesis of different mercapto triazoles. Hydrolysis by aqueous KOH of ((2*E*,4*E*)-5-(benzo[*d*][1,3]dioxol-5-yl)-1-(piperidin-1-yl)penta-2,4-dien-1-one) (**106**) gave 5-(benzo[*d*][1,3]dioxol-5-yl)penta-2,4-dienoic acid (**107**). (5-(Benzo[*d*][1,3]dioxol-5-yl)penta-2,4-dienehydrazide (**108**) was obtained after reacting the acid (**107**) with oxalyl chloride followed by hydrazine hydrate. Compounds **109a**–**n** were prepared from reaction of the acid hydrazide **108** using different isothiocyanates. Basic hydrolysis of **109a**–**n** efficiently gave the desired 3-((1*E*,3*E*)-4-(benzo[*d*][1,3]dioxol-5-yl)buta-1,3-dien-1-yl)-4-substituted-1*H*-1,2,4-triazole-5(4*H*)- thiones **110a**–**n** in 32–51% yields (Scheme 28) [18]. The best trypanocidal activity was noted in case of **110g** on proliferative forms of *Trypanosoma cruzi* [18].

**Scheme 29:**
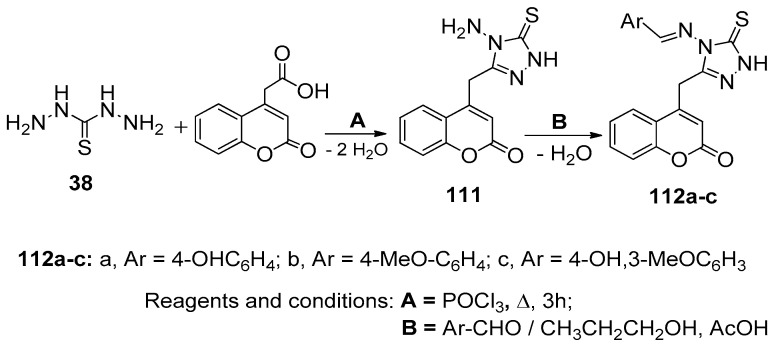
Synthesis of chromen-2-ones derived by triazolethiones **112a**–**c**.

Reaction of 2-(coumarin-4-yl)acetic acid with thiocarbohydrazide (**38**) in refluxing phosphoryl chloride yielded the target 4-((4-amino-5-thioxo-4,5-dihydro-1*H*-1,2,4-triazole-3-yl)methyl)-2*H*-chromen-2-one (**111**) in 80% yield. Condensation of **111** with various aromatic aldehydes yielded (*E*)-4-((4-(benzylideneamino)-5-thioxo-4,5-dihydro-1*H*-1,2,4-triazole-3-yl)methyl)-2*H*-chromen-2-ones **112a**–**c** (Scheme 29) [37]. The synthesized compounds were evaluated *in vitro* as anticancer agents in the human colon cancer (HCT 116) cell line. Compound **112c** showed high anticancer activity (relative potency >50%) with IC_50_ value of 4.363 μM compared to the potent anticancer drug doxorubicin, whereas compound **112a** displayed moderate anticancer activity with IC_50_ values 18.76 μM. The molecular docking studies of the active compounds revealed that these compounds might act via inhibition of tyrosine kinases (CDK2) [37].

**Scheme 30:**
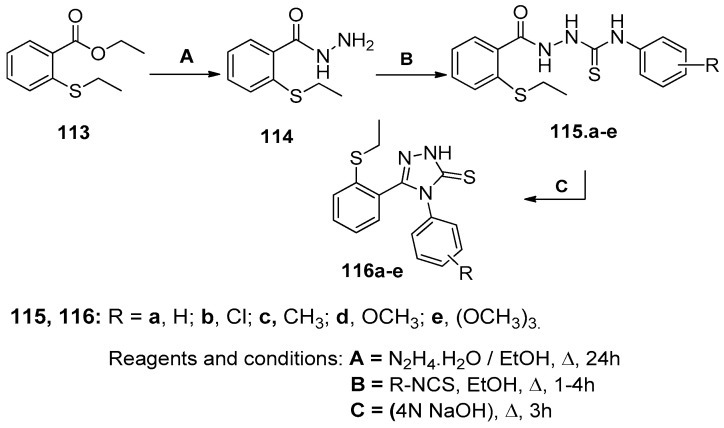
Synthesis of triazolethiones **116a**–**e**.

Compound 2-(ethylthio)benzohydrazide (**114**) was obtained by refluxing ethyl 2-(ethylthio)benzoate (**113**) with hydrazine hydrate. Then, subjecting **114** with various aryl isothiocyanates yielded the thiosemicarbazides **115a**–**e**. Compounds **115a**–**e** were then cyclized to *N*-substituted triazolethiones **116a**–**e** in 69–75% yields (Scheme 30) [65]. Compounds **115a**,**b** and **116a**, were effectively used as antioxidant agents with IC_50_ values of 1.08, 0.74, and 0.22 μg/mL, respectively, compared to gallic acid (IC_50_ = 1.2 μg/mL) [65].

**Scheme 31:**
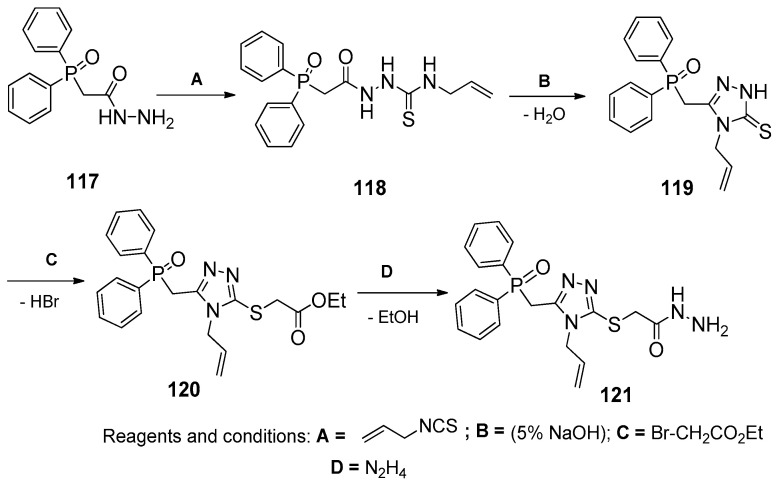
Synthesis of triazolethione **121**.

Phosphorylated triazolethione **119** was formed in 90% yield from the cyclization process of *N*-allyl-2-(2-(diphenylphosphoryl)acetyl)hydrazinecarbothioamide **118** (obtained from 2-(diphenylphosphoryl)acetohydrazide (117) with allyl isothiocyanate) in basic media (5%) NaOH [42]. Moreover, ethyl 2-((4-allyl-5-((diphenylphosphoryl)-methyl)-4*H*-1,2,4-triazole-3-yl)thio)acetate (**120**) was synthesized in 68% yield by reacting **119** with ethyl bromoacetate. Hydrazinolysis of compound **120** gave 2-((4-allyl-5-((diphenylphosphoryl)methyl)-4*H*-1,2,4-triazole-3-yl)thio)acetohydrazide (**121**) in 43% yield (Scheme 31) [42].

**Scheme 32:**
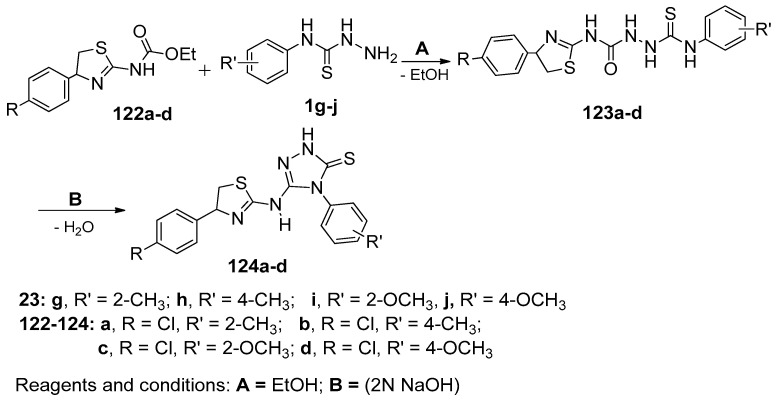
Synthesis of 2,4-triazole-5(4*H*)-thiones **124a**–**d**.

Incorporating of triazolethiones into thiazole ring was the optimum pharmacophore model for anticonvulsant activities, thus condensation of ethyl thiazol-2-ylcarbamates **122a**–**d** with 4-substituted thiosemicarbazides **23c**–**f** afforded the corresponding hydrazinecarbothioamides **123a**–**d**. Cyclization of the latter with aqueous sodium hydroxide gave 4-substituted phenyl-3-((4-aryl-4,5-dihydrothiazol-2-yl)amino)-1*H*-1,2,4-triazole-5(4*H*)-thiones **124a**–**d** in 62–84% yields [66] (Scheme 32). The obtained compounds were screened for their anticonvulsant activity and showed that compounds **124d** and **124e** had a significant anticonvulsant activity compared with the standard drugs [66].

**Scheme 33:**
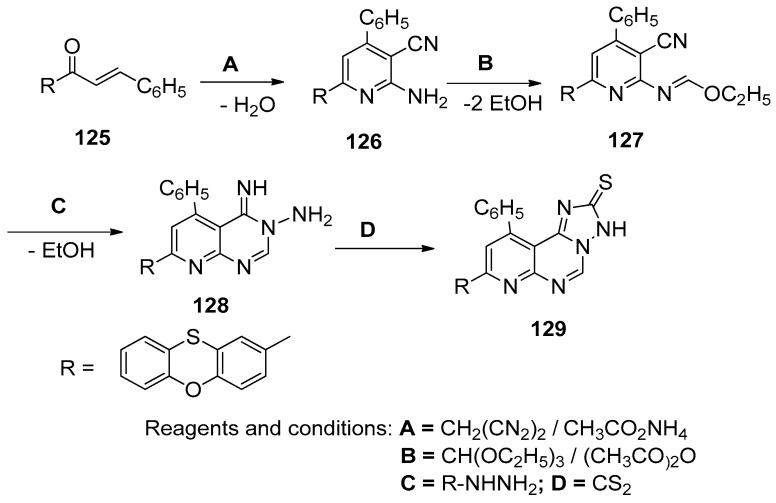
Synthesis of [1,2,4]triazolo-pyrimido[4-*b*]benzo[5,6][1,4]oxathiino[3,2-*g*]quinoline-2(3*H*)-thiones **129**.

The condensation of (*E*)-1-(phenoxathiin-2-yl)-3-phenylprop-2-en-1-one (**125**) with malononitrile afforded 2-amino-6-(phenoxathiin-2-yl)-4-phenylnicotinonitrile (**126**). In addition, compound **126** reacted with triethyl orthoformate in acetic anhydride to give formimide **127**, which upon hydrazinolysis with phenyl hydrazine yielded 4-imino-7-(phenoxathiin-2-yl)-5-phenylpyrido[2-*d*]pyrimidin-3(4*H*)-amine (**128**). The reaction of the latter with CS_2_ gave 16-phenyl-[1,2,4]triazolo-pyrimido[4-*b*]benzo[5,6][1,4]oxathiino[3,2-*g*]quinoline-2(3*H*)-thiones **129** (Scheme 33) [50].

**Scheme 34:**
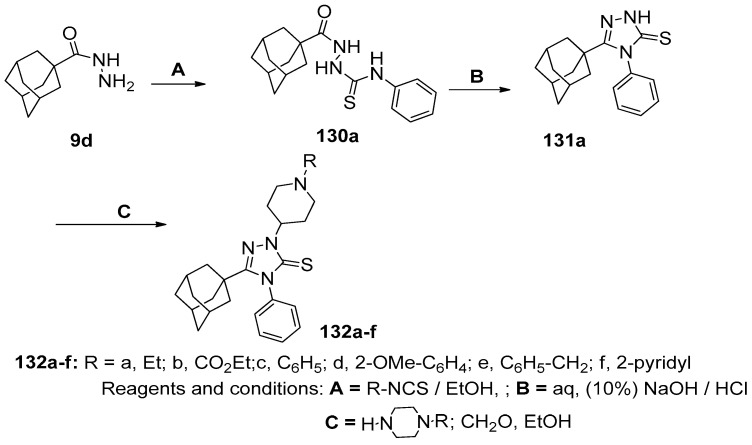
Synthesis of 1,2,4-triazole-5(4*H*)-thiones **132a**–**f**.

2-(Adamantanyl-1-carbonyl)-*N*-phenylhydrazinecarbothioamide (**130**) was synthesized from the reaction of adamantane-1-carbohydrazide (**9d**) with phenyl isothiocyanate. Thereafter, 3-(adamantan-1-yl)-4-phenyl-1*H*-1,2,4-triazole-5(4*H*)-thione **131a** was synthesized *via* basic hydrolysis of **130a** with NaOH. Then, 3-(adamantan-1-yl)-1-((piperidin-4-yl)methyl)-4-phenyl-1*H*-1,2,4-triazole-5(4*H*)-thiones **132a**–**f** were obtained from the reaction of compound **131a** with 1-substituted piperazine and formaldehyde solution [34] (Scheme 34). The synthesized *N*-Mannich bases of triazolethiones **132b**–**f** screened against Gram-positive and -negative bacteria in addition to some pathogenic fungus (*Candida albicans*) revealed that they had potent antibacterial activity [34].

**Scheme 35:**
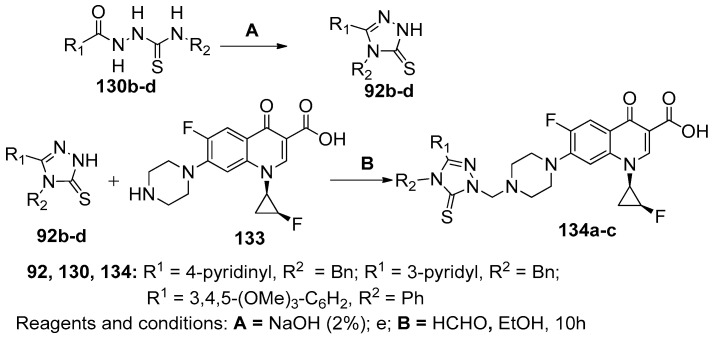
Synthesis of 1,2,4-triazole-thiones of 1-[(1*R*,2*S*)-2-fluorocyclopropyl]-CPFX **134a**–**c**.

Hydrolysis of disubstituted hydrazinecarbothioamides **130b**–**d** with aqueous sodium hydroxide gave 1,2,4-triazole-5-thiones **92b**–**d**. Mannich reaction of 1,2,4-triazole-5-thiones **92b**–**d**, 1-[(1*R*,2*S*)-2-fluorocyclopropyl]CPFX (**133**) and formaldehyde afforded 1-[(1*R*,2*S*)-2-fluorocyclopropyl]CPFX-1,2,4-triazole-5-thiones **134a**–**c** in 52–57% yields (Scheme 35) [67].

Antibacterial activity against different pathogens showed that all of the CPFX-1,2,4-triazole-5-thiones **134a**–**c** were more potent than the parent 1-[(1*R*,2*S*)-2-fluorocyclopropyl]-CPFX **133** and comparable to ciprofloxacin and levofloxacin against the majority of the tested pathogens. Moreover, the anti-Gram negative bacterial activity of **134a**–**c** was far more potent than the reference named Vancomycin (VAN) [67].

**Scheme 36:**
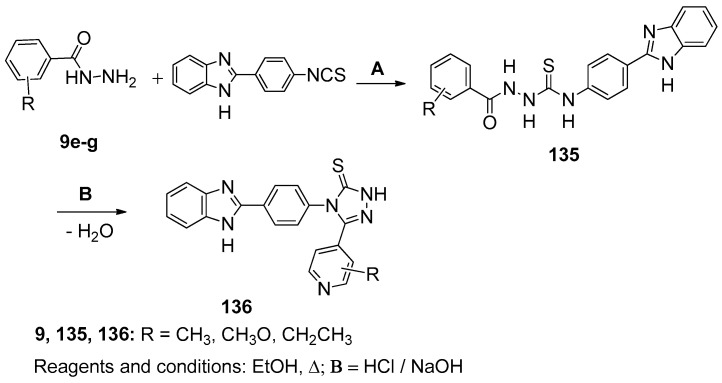
Synthesis of 1,2,4-triazole-thiones **136**.

The reaction of acid hydrazide **9e**–**g** and 2-(4-isothiocyanatophenyl)-1*H*-benzo[*d*]imidazole gave *N*-(4-(1*H*-benzimidazol-2-yl)phenyl)-2-benzoylhydrazine-carbothioamides **135**. Ring closure of compound **135** in acidic media afforded 1,2,4-triazole-5-thiones **136** [28] in 55–64% yields (Scheme 36). Screening of the obtained compounds for antibacterial and antifungal activities showed that some of these compounds exhibited good antibacterial and antifungal activities [28]. 

**Scheme 37:**
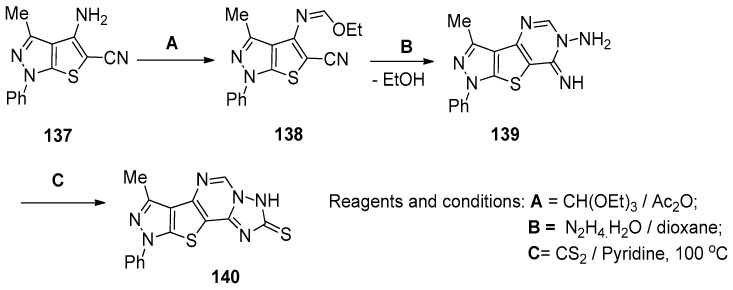
Synthesis of 1,2,4-pyrazolothieno[2,3-*e*][1,2,4]triazolo[1,5-*c*]pyrimidine-2-thione **140**.

Condensation of 4-amino-3-methyl-1-phenyl-1*H*-thieno[2,3-c]pyrazole-5-carbonitrile (**137**) with triethyl orthoformate in the presence of acetic anhydride as catalyst gave (*Z*)-ethyl *N*-(5-cyano-3-methyl-1-phenyl-1*H*-thieno[2,3-*c*]pyrazol-4-yl)formimidate (**138**). Hydrazinolysis of **138** with hydrazine hydrate yielded 7-imino-3-methyl-1-phenyl-1*H*-pyrazolothieno[3,2-*d*]pyrimidin-6(7*H*)-amine (**139**), whereas cyclization of pyrazolothienopyrimidines **139** with CS_2_ afforded 7-methyl-9-phenyl-3,9-dihydro-2*H*-pyrazolothieno[2,3-*e*][1,2,4]triazolo[1,5-*c*]pyrimidine-2-thione (**140**) in 35% yield (Scheme 37) [54]. 

**Scheme 38:**
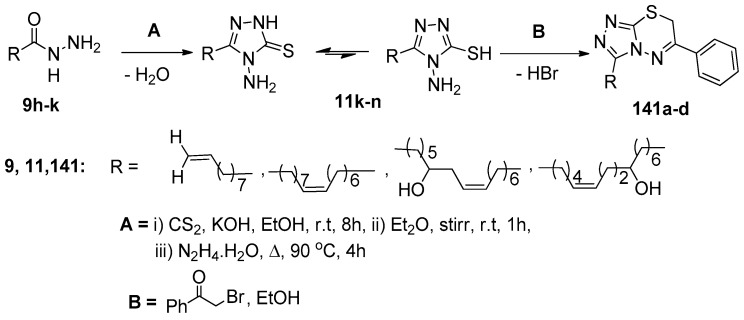
Synthesis of fused triazolothiadiazines **141a**–**d**.

The cyclization reaction of longer alkenyl/hydroxyl alkenyl acid hydrazides **9h**–**k** CS_2_/KOH followed by treatment with hydrazine hydrate yielded the corresponding 4-amino-3-substituted-1*H*-1,2,4-triazole-5(4*H*)-thiones **11k**–**n**. 1,2,4-Triazolothiadiazines **141a**–**d** were directly obtained from the reaction of amino triazolethiones **11k**–**n** with phenacyl bromide in 62–90% yields (Scheme 38). *In vitro* screening of anticancer activity against three different cell lines, i.e., human hepatocellular carcinoma (Hep3B), human breast adenocarcinoma (MCF7), and human cervical carcinoma (HeLa), towards triazolethione and triazolothiadiazines showed that the nature of the long-chain on third position affected the potency of these drugs. Besides, fused triazolothiadiazines **141a**–**d** were found to be potential anticancer agents [68].

**Scheme 39:**
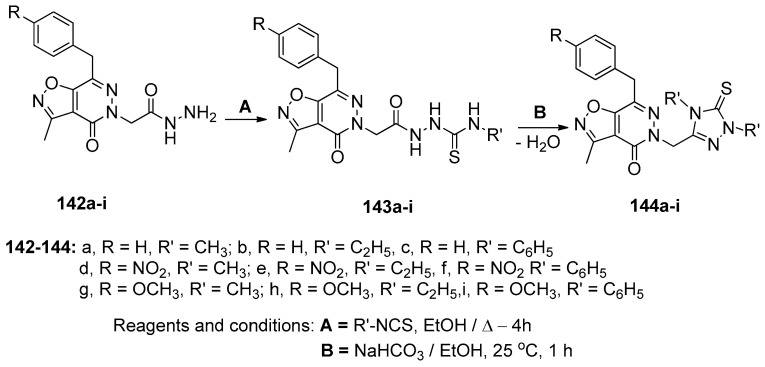
Synthesis of triazolethiones **144a**–**i**.

Similarly, the reaction of acid hydrazides **142a**–**i** with isothiocyanates afforded the acylhydrazides **143a**–**i** (Scheme 39). The triazolethiones **144a**–**i** were then obtained in 62–90% yields from the reaction of thiosemicarbazides **143a**–**i** in NaHCO_3_ in ethanol (Scheme 39) [69]. The 1,2,4-triazolethione **144g** was found to be the best anti-inflammatory nucleus *via* inhibiting both COX-2 (IC_50_ = 2.1 μM) and 5-LOX (IC_50_ = 2.6 μM) enzymes, and this was supported *via* enzyme-ligand molecular modeling (docking studies), which gave favorable binding interactions in both COX-2 and 5-LOX active sites. It also has a superior gastrointestinal safety profile (ulcer index = 0.25) compared to the reference drug [69].

**Scheme 40:**
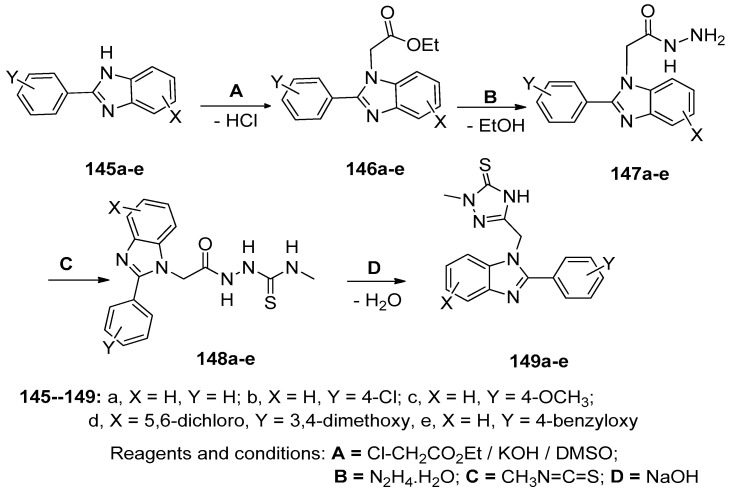
Synthesis of triazolethiones **149a**–**e**.

Interestingly, esterification of 1*H*-benzimidazoles **145a**–**e** with ethyl chloroacetate gave ethyl 2-(2-phenyl-1*H*-benzo[*d*]imidazol-1-yl)acetates **146a**–**e**, which upon reacting with hydrazine hydrate gave the acid hydrazide **147a**–**e**. Also, *N*-methyl-2-(2-(2-phenyl-1*H*-benzo[*d*]imidazol-1-yl)acetyl)hydrazinecarbothioamides **148a**–**e** were formed by the reaction **147a**–**e** with methyl isothiocyanate. Ring closure of hydrazine-carbothioamides **148a**–**e** with aqueous NaOH afforded the biologically active triazolethiones **149a**–**e** in 51–64% yields, with significant antioxidant properties (Scheme 40) [70].

**Scheme 41:**
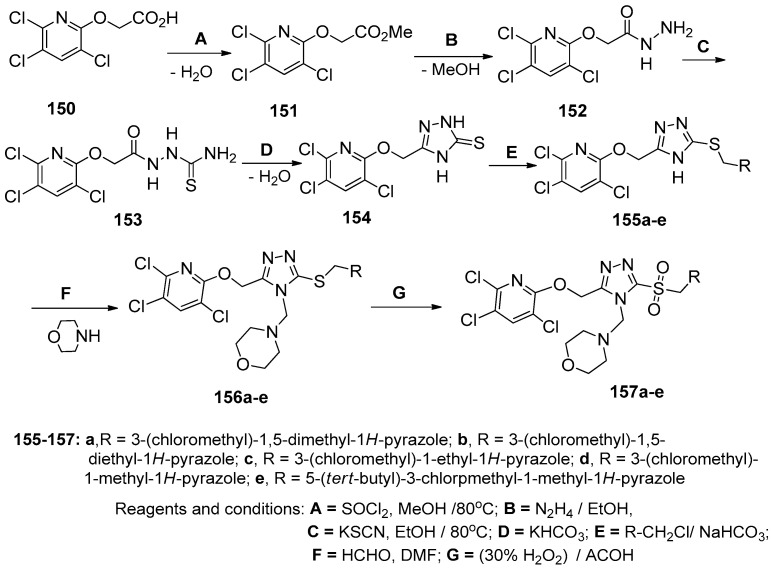
Synthesis of 3,4,5-trisubstituted-1,2,4-triazoles **157a**–**e**.

Reaction of 2-((3,5,6-trichloropyridin-2-yl)oxy)acetic acid (**150**) with thionyl chloride gave compound **151**. Additionally, the treatment of compound **151** with hydrazine hydrate gave (3,5,6-trichloropyridin-2-yl)hydrazine-carboxylate (**152**). When the acid hydrazide **152** was then subjected to aqueous potassium thiocyanate, 2-(2-((3,5,6-trichloropyridin-2-yl)oxy)acetyl)hydrazine-carbothioamide (**153**) was obtained. Cyclization of **153** in basic media afforded 1*H*-1,2,4-triazole-5(4*H*)-thione **154**) [71]. On the other side, various *S*-alkylated products **155a**–**e** were obtained *via* reacting substituted benzyl chlorides with triazolethiones **154**. However, the reaction of morpholine, formaldehyde, and compound **155a**–**e** gave the *N*-alkylated morpholino-triazolethiones **156a**–**e**, which on oxidation with H_2_O_2_ in acidic media gave 3,4,5-trisubstituted-1,2,4-triazoles **157a**–**e** in 54–69% yields [71]. The synthesized compounds **157a**–**e** screened for their antimicrobial activity revealed that **157c** exhibited better antibacterial and antifungal activities than the other compounds (Scheme 41) [71].

**Scheme 42:**
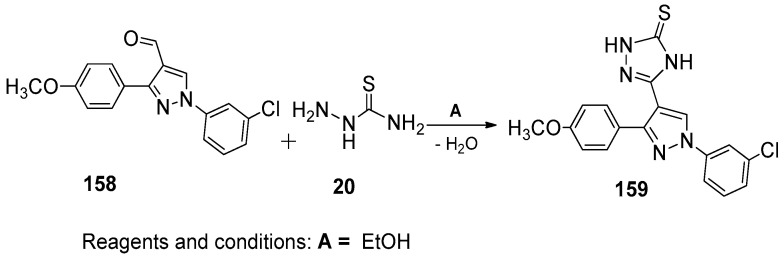
Synthesis of triazolethione **159**.

On refluxing of 1-(3-chlorophenyl)-3-(4-methoxyphenyl)-1*H*-pyrazole-4-carbaldehyde (**158**) with thiosemicarbazide (**20**), the reaction proceeded to give the corresponding 3-(1-(3-chlorophenyl)-3-(4-methoxyphenyl)-1*H*-pyrazol-4-yl)-1*H*-1,2,4-triazole-5(4*H*)-thione (**159**) (Scheme 42). The bioassay of triazolethione **159** showed that it has significant anti-inflammatory activity [72].

**Scheme 43:**
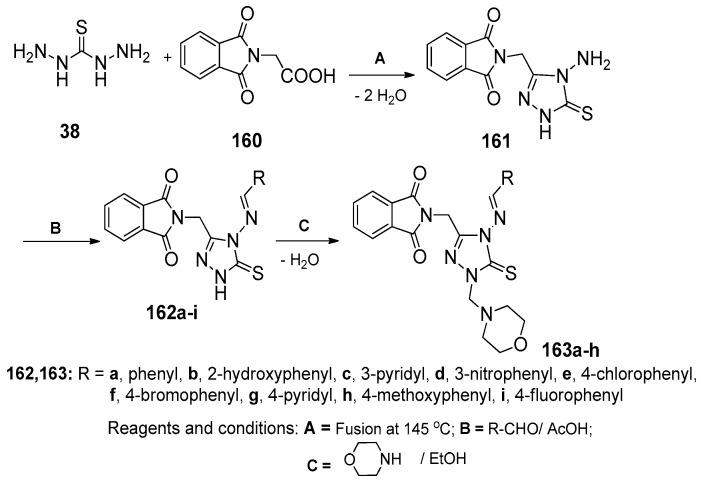
Synthesis of triazolethiones **163a**–**h**.

Fusion of thiocarbohydrazide (**38**) with 2-(1,3-dioxoisoindolin-2-yl)acetic acid (**160**) gave the 2-((4-amino-5-thioxo-4,5-dihydro-1*H*-1,2,4-triazole-3-yl)methyl)-isoindoline-1,3-dione (**161**) in 69% yield. The synthesis of triazolethione Schiff bases **162a**–**i** was achieved, in 35–66% yield, by refluxing of **161** with different aromatic aldehydes. Besides, Mannich bases **163a**–**h** were easily obtained from the reaction of Schiff bases **162a**–**i** with formaldehyde and morpholine in 39–82% yields (Scheme 43) [73]. The antimicrobial bioassay of these compounds **163a**–**h** showed that antimicrobial activity was increased by introducing azomethine group and also by the addition of morpholine group leading to prospective antimicrobial agents with **163a**–**b**, **163e**–**f**, and **163h** [73]. 

### Synthesis of Spiro-1,2,4-triazolethiones

**Scheme 44:**
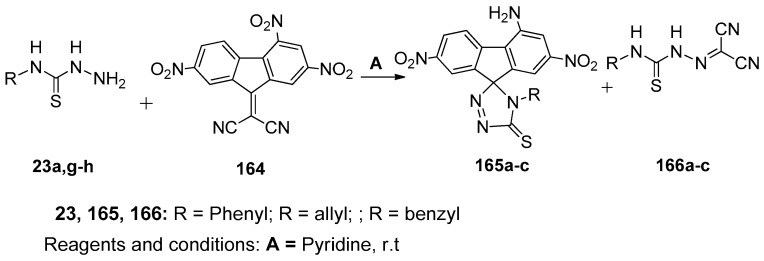
Synthesis of spiro-triazolethiones **165a**–**c**.

Spiro-triazolethiones **165a**–**c** were also obtained from donor–acceptor interactions in such as in case of reacting hydrazinecarbothioamides **23a**,**g**–**h** with trinitrofluorenone (DTF) **164** in addition to 1,4-disubstituted hydrazine-carbothioamides **166a**–**c** in 23–25% yields, respectively (Scheme 44) [74].

**Scheme 45:**
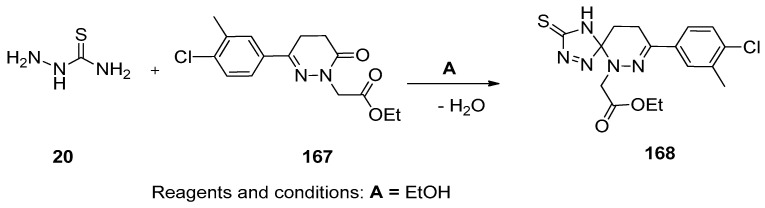
Synthesis of ethyl 2-(substituted phenyl)-3-thioxo-1,2,4,6,7-pentaazaspiroacetate (**168**).

It was reported that thiosemicarbazide (**20**) reacted with dihydropyridazin-1(4*H*)-yl)acetate to get ethyl 2-(substituted phenyl)-3-thioxo-1,2,4,6,7-pentaazaspiroacetate (**168**) [75] (Scheme 45). 

**Scheme 46:**
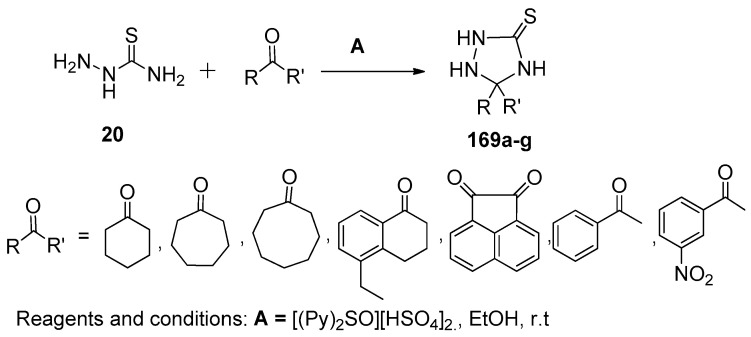
A series of spiro-1,2,4-triazole-3-thiones **169a**–**g**.

Patil et al. [32] reported the synthesis of a series of spiro-1,2,4-triazole-3-thiones **169a**–**g** varied from good to excellent yields (55–95%) from thiosemicarbazide (**20**) and different cyclic ketones using different catalysts, the most effective catalyst was 1,1′-sulfinyldipyridinium bis(hydrogen sulfate) ionic liquid which gave high yield and short reaction time in alcohol at room temperature (Scheme 46).

**Scheme 47:**
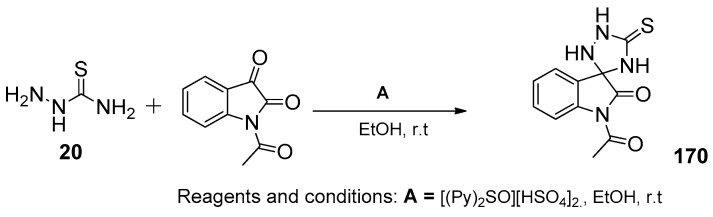
Synthesis of 1-acetyl-5′-thioxospiro[indoline-3,3′-[1,2,4]triazolidin]-2-one (**170**).

The synthesis of 1-acetyl-5′-thioxospiro[indoline-3,3′-[1,2,4]triazolidin]-2-one (**170**), in 87% yield, was achieved by the reaction of 1-acetylindoline-2,3-dione with thiosemicarbazide **20** catalyzed by acidic ionic liquid (Scheme 47). Compound **170** showed good antibacterial activity [30]. 

**Scheme 48:**
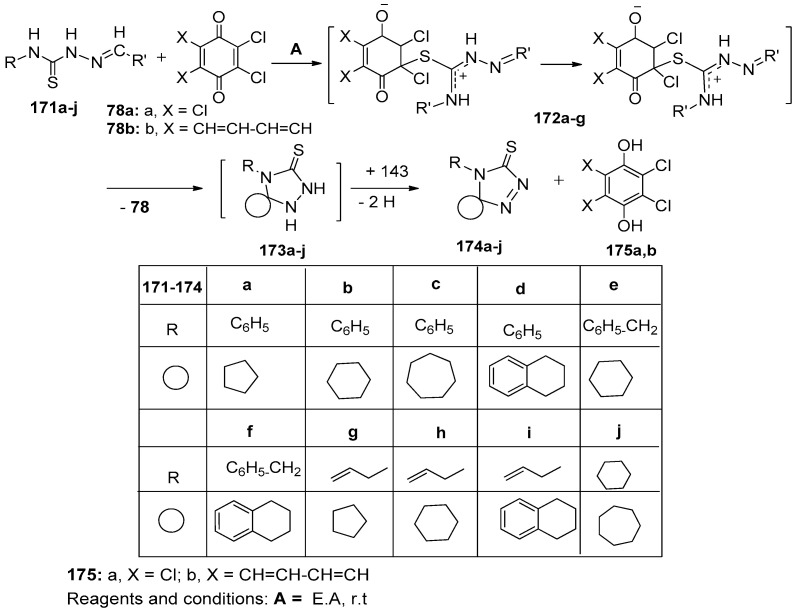
Synthesis of spiro-1,2,4-triazole-3-thiones **174a**–**j**.

Reactions of thiosemicarbazides **171a**–**j** with various π-acceptors such as benzo- or naphthoquinones **78a**,**b** led to different fused heterocyclic rings [76]. However, spiro-1,2,4-triazole-3-thiones **174a**–**j** were obtained from the reaction of cycloalkanone-thiosemicarbazides **171a**–**j** with benzo- or naphthoquinones **78a**,**b** in 80–85% yields (Scheme 48) [76].

**Scheme 49:**
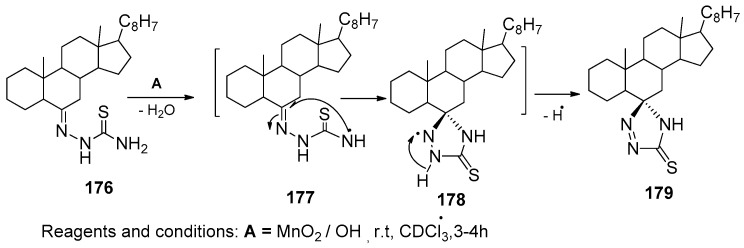
Synthesis of spirotriazolethione **179**.

**Scheme 50:**
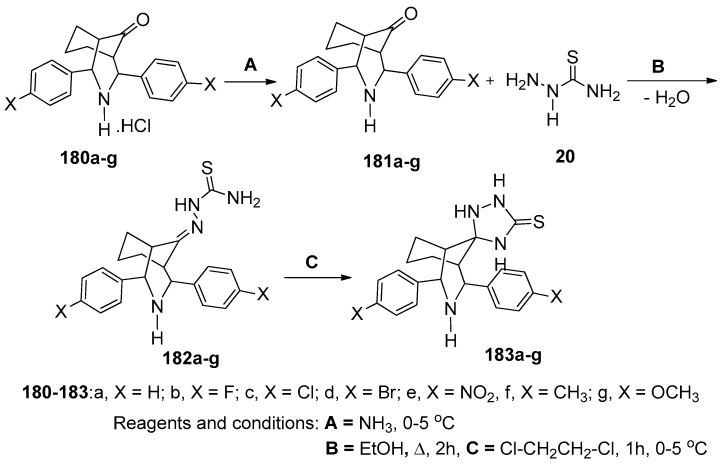
Synthesis of spiro-triazolethiones **183a**–**g**.

Substituted thiosemicarbazone **176** was cyclized to the corresponding spirotriazolethione **179** in 70–82% yields through the intermediates **177** and **178** (Scheme 49) [77]. 

(1*R*,2*R*,4*R*,5*S*)-2,4-Disubstituted phenyl-3-azabicyclo[3.3.1]nonan-9-one hydro-chlorides **180a**–**g** reacted with ammonia to give (1*R*,2*R*,4*R*,5*S*)-2,4-disubstituted phenyl-3-azabicyclo[3.3.1]nonan-9-ones **181a**–**g** (Scheme 50) [31]. Condensation of compounds **181a**–**g** with thiosemicarbazide **20** afforded compounds **182a**–**g**, which upon cyclization in the presence of *m*-chlorobenzaldehyde efficiently gave spiro-1,2,4-triazoline-3′-thiones **183a**–**g** in 50–70% yields (Scheme 50). Screening of these spiro-triazolethiones **183a**–**g** for antibacterial and antifungal activities showed that compounds **183b**–**e** had excellent antifungal activity against all the tested microorganisms. However, compounds **183d**,**e** showed excellent antibacterial activity against *β-H. streptococcus*. Besides, compounds **183b**–**c**,**e** showed varied activities toward the tested Gram-positive and -negative strains [31].

**Scheme 51:**
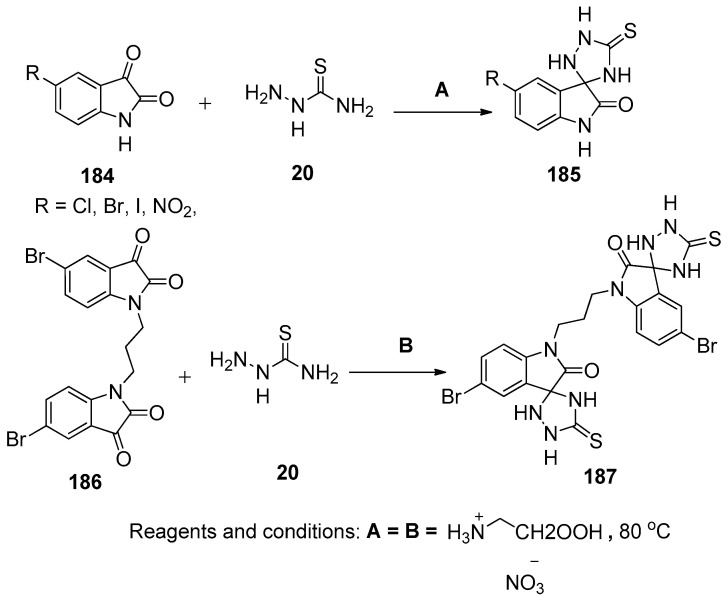
Synthesis of bis-spirotriazolethione **187**.

5-Substituted-5′-thioxospiro[indoline-3,3′-[1,2,4]triazolidin]-2-ones **185a**–**d** (83–89% yields) successfully were obtained from the reaction of different 5-substituted indoline-2,3-diones **184a**–**d** with **20** in water and catalyzed by using glycine nitrate. In the same manner, bis-spirotriazolethione **187** was synthesized from the reaction of 1,1′-(propane-1,3-diyl)bis(5-bromoindoline-2,3-dione) **186** with **20** in 89% yield (Scheme 51) [78]. 

**Scheme 52:**
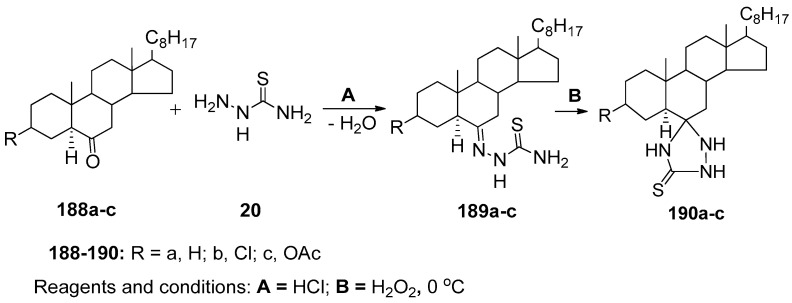
Synthesis of spiro-[cyclopenta[*a*]phenanthrene-6,3′-1,2,4triazolidine]-5′-thiones **190a**–**c**.

Condensation of gonanone derivatives **188a**–**c** with **20** in acidic media gave the corresponding thiosemicarbazones **189a**–**c**. Oxidative cyclization of thiosemicarbazones **189a**–**c** with hydrogen peroxide yielded the corresponding (5*S*)-10,13-dimethyl-17-octylhexadecahydrospiro-[cyclopenta[*a*]phenanthrene-6,3′-1,2,4triazolidine]-5′-thiones **190a**–**c** in 66–78% yields (Scheme 52) [79].

**Scheme 53:**
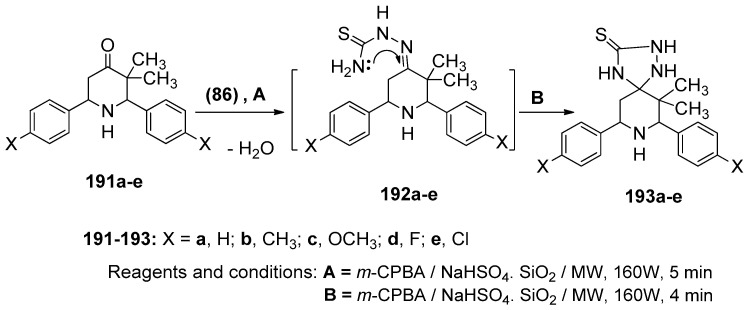
Synthesis of tetrazaspiro[4.5]decane-3-thiones **193a**–**e**.

Microwave irradiation was used as an efficient method to get good yields with a shorter time than the classical method for the synthesis of 6,6-dimethyl-phenyl-1,2,4,8-tetrazaspiro[4.5]decane-3-thiones **193a**–**e** *via* formation of thiosemicarbazone intermediates **192a**–**e**, which was obtained from the reaction of 3,3-dimethyl-phenylpiperidin-4-ones **191a**–**e** with **20** (Scheme 53) [80].

**Scheme 54:**
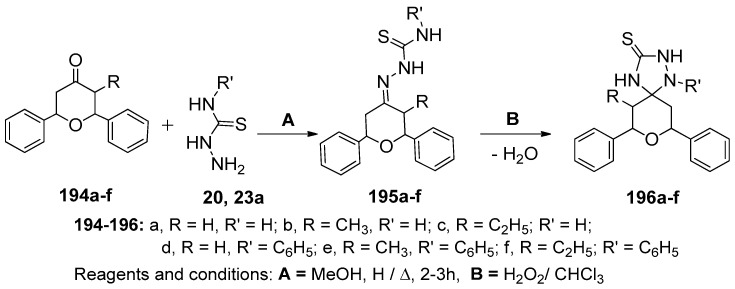
Synthesis of 1,2,4-triazaspiro[4.5]decane-3-thiones **196a**–**f**.

Similarly, the reaction of 3-alkyl-2,6-diphenylpyran-4-ones **194a**–**f** with **20** or 4-phenyl hydrazinecarbothioamides **23** gave thiosemicarbazones **195a**–**f**. Oxidative cyclization of **195a**–**f** with hydrogen peroxide led to the expected 7,9-diphenyl-8-oxa-1,2,4-triazaspiro[4.5]decane-3-thiones **196a**–**f**. The synthesized compounds were tested for antimicrobial activity against *Staphylococcus aureus*, *Escherichia coli*, *Pseudomonas aeruginosa*, *Salmonella typhi*, *Aspergillus flavus*, *Aspergillus niger*, *Candida albicans*, and *Rhizopus* sp. Compounds **196e** and **196f** showed potent antibacterial activity against *E. coli* and *S. typhi*. However, compounds **196d** and **196f** were potent against *Rhizopus* sp., whereas compound **196e** gave significant antifungal activity against *Aspergillus flavus* (Scheme 54) [81].

**Scheme 55:**
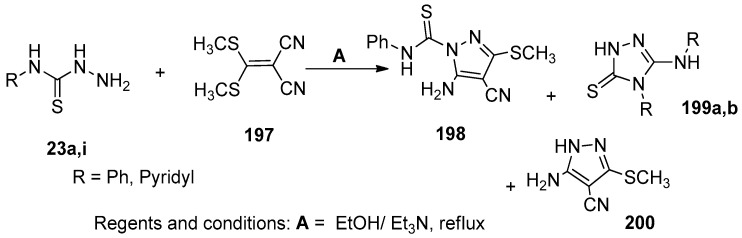
Formation of triazolothiones **199a**,**b**.

Aly et al. [82] reported that reaction of equal equivalents of both *N*-substituted hydrazinecarbothioamides **23a**,**i** with 2-(bis(methylthio)methylene)malononitrile (**197**) in dry ethanol catalyzed by few drops of Et_3_N for 3 h afforded a colorless precipitate of 5-amino-4-cyano-3-(methylthio)-*N*-phenyl-1*H*-pyrazole-1-carbothioamide (**198**) as the major product in 65% yield together with 3,4-disubstituted amino-1*H*-1,2,4-triazole-5(4*H*)-thiones **199a**,**b** and pyrazole carbonitrile **200** as minor products (Scheme 55) [82].

## 3. Reactions of 1,2,4-Triazolethiones

### 3.1. Synthesis of Open-Chain Compounds

**Scheme 56:**
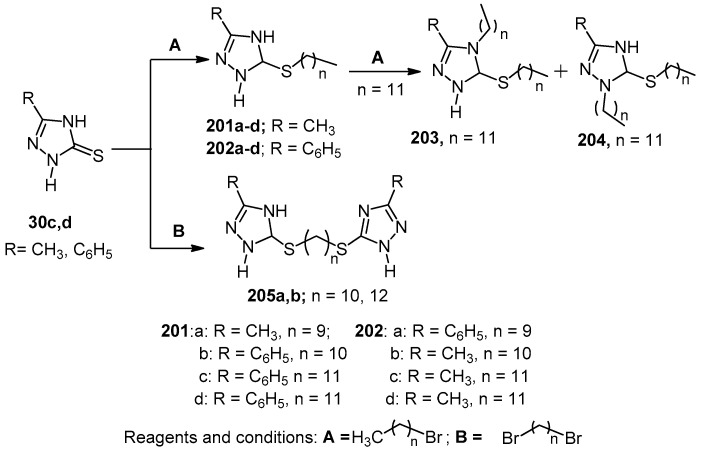
Synthesis of bis-1,2,4-triazoles **205a**,**b**.

The synthesis of mono and bipolar surfactants **201a**–**d** and **202a**–**d** was achieved by the reaction of 3-methyl-1*H*-1,2,4-triazole-5(4*H*)-thione (**30c**) and 3-phenyl-1*H*-1,2,4-triazole-5(4*H*)-thione (**30d**) with various alkyl bromides. However, in the case of *n*-dodecyl bromide, a mixture of two isomers **203** and **204** was obtained from the reaction of **201a**–**d** and **202a**–**d** with another molecule of alkyl bromide. In addition, bis-1,2,4-triazoles **205a**,**b** were obtained from the reaction of linear dibromoalkanes with 3-methyl-1*H*-1,2,4-triazole-5(4*H*)-thione (**30c**) (Scheme 56) [83]. 

**Scheme 57:**
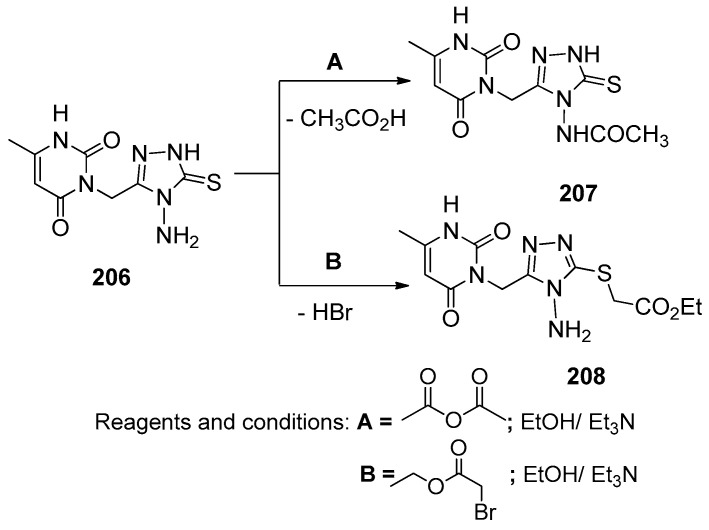
Synthesis of 1,2,4-triazole-3-yl)thio)acetate (**208**).

Actylation of 3-substituted aminotriazolethione **206** by acetic anhydride afforded 4-substituted-1,2,4-triazole-5-thione **207** in 52% yield (Scheme 57). Additionally, ethyl 2-((4-amino-5-((6-methyl-2,4-dioxo-1,2,3,4-tetrahydropyridin-3-yl)methyl)-4*H*-1,2,4-triazole-3-yl) thio)acetate (**208**) can be obtained in 50% yield upon reacting ethyl 2-bromoacetate with compound **206** (Scheme 57) [84].

**Scheme 58:**
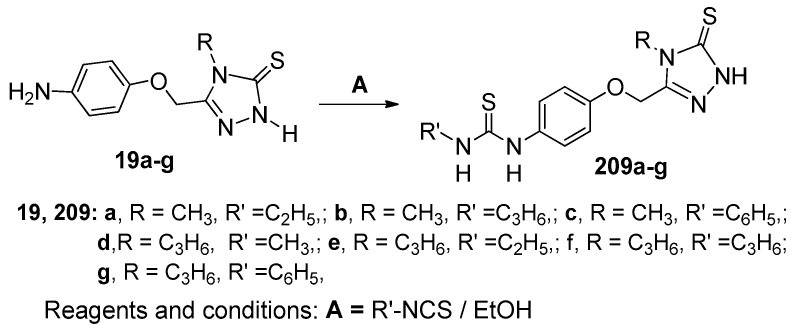
Synthesis of 1,2,4-triazolethiones **209a**–**g**.

Reaction of various 1,2,4-triazolethiones **19a**–**g** with alkyl or aryl isothiocyanates gave 1-substituted-3-(4-((4-substituted-5-thioxo-4,5-dihydro-1*H*-1,2,4-triazole-3-yl)-methoxy)phenyl)thio-ureas **209a**–**g** (Scheme 58). All the synthesized compounds **209a**–**g** evaluated against antiviral, anti-HIV, and anti-tuberculosis activity showed that compound **209g** was the most active one with 79% inhibition against *Mycobacterium tuberculosis* H37Rv and also gave moderate protection against Coxsackievirus B4 with an MIC value of 16 mg/mL and a selectivity index of 5 [21]. 

**Scheme 59:**
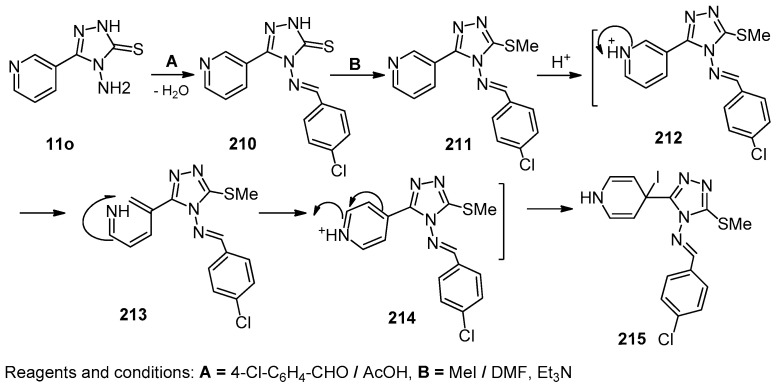
Synthesis of trisubstituted 1,2,4-triazole **215**.

Condensation of 4-amino-5-(pyridin-3-yl)-1,2,4-triazolidine-3-thione (**11o**) with 4-chlorobenzaldehyde yielded 4-chlorobenzylideneamino-5-(pyridin-3-yl)-1,2,4-triazolidine-3-thione (**210**). (*E*)-4-Chloro-benzylideneamino-5-(methylthio)-3-(pyridin-3-yl)-1,2,4-triazole **211** was synthesized form the reaction of **210** with methyl iodide. Finally, the trisubstituted 1,2,4-triazole **215** was synthesized in presence of iodide anion through the intermediates **212**–**214** (Scheme 59) [85]. 

**Scheme 60:**
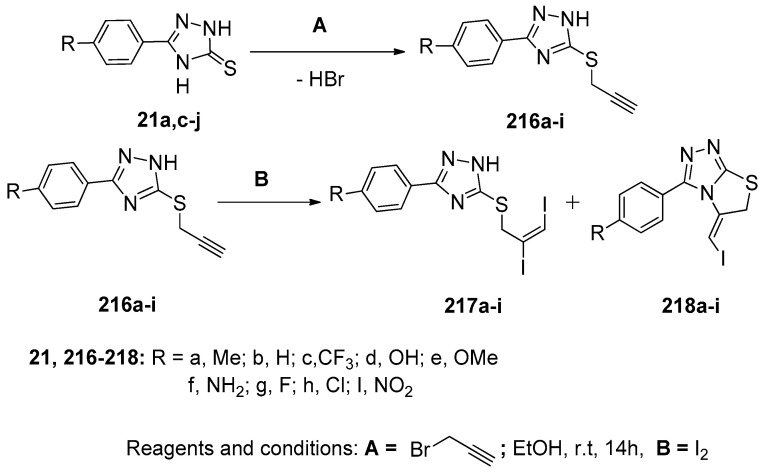
Synthesis of 3-(substituted phenyl)-1,2,4-triazoles **217a**–**i**.

The reaction of propargyl bromide with 5-(substituted phenyl)-1,2,4-triazolidine-3-thiones **21a**,**c**–**j** yielded 3-substituted-5-(prop-2-yn-1-ylthio)-4,5-dihydro-1*H*-1,2,4-triazoles **216a**–**i** in 62–77% yields. Besides, the reaction of compounds **216a**–**i** with iodine afforded (*E*)-5-((2,3-diiodoallyl)thio)-3-(substituted phenyl)-1,2,4-triazoles **217a**–**i** in 75–92% yields and traces of thiazolotriazoles **218a**–**i** in 44–59% yields (Scheme 60) [86].

**Scheme 61:**
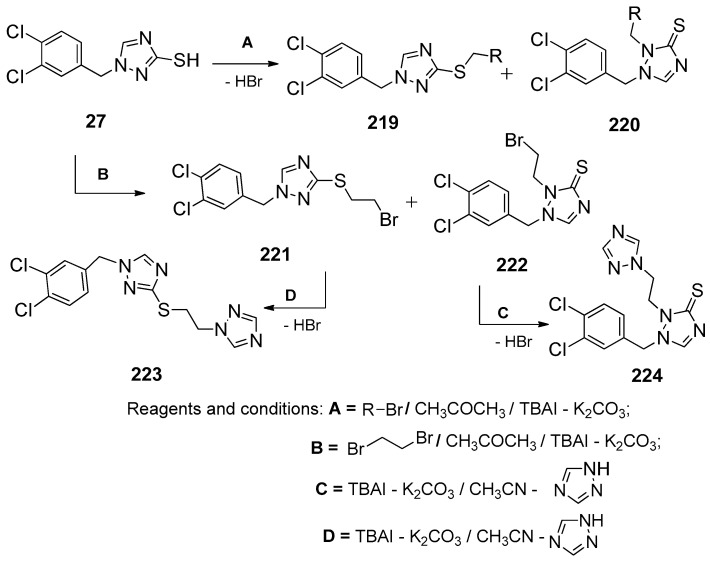
Synthesis of 1,2,4-triazol-1-yl)ethyl)thio)-1-(3,4-dichlorobenzyl)-1*H*-1,2,4-triazole (**224**).

The synthesis of *S*- and *N*-alkylated products of triazolethiones **219** and **220** was achieved by the reaction of 1-(3,4-dichlorobenzyl)-1*H*-1,2,4-triazole-3-thiol (**27**) with 1-bromooctane in basic media using tetrabutylammonium bromide (TBAB) as a catalyst in acetone for several minutes. Besides, 3-((2-bromoethyl)thio)-1-(3,4-dichlorobenzyl)-1*H*-1,2,4-triazole (**221**) and 2-(2-bromoethyl)-1-(3,4-dichlorobenzyl)-1*H*-1,2,4-triazole-3(2*H*)-thione (**222**) were prepared from the reaction of **27** with 1,2-dibromoethane as mentioned before. On the other hand, reacting **222** with 1,2,4-triazole afforded 2-(2-(1*H*-1,2,4-triazol-1-yl)ethyl)-1-(3,4-dichlorobenzyl)-1*H*-1,2,4-triazole-3(2*H*)-thione (**223**), whereas reaction of 1,2,4-triazole with **221** gave 3-((2-(1*H*-1,2,4-triazol-1-yl)ethyl)thio)-1-(3,4-dichlorobenzyl)-1*H*-1,2,4-triazole (**224**) [87]. The screening of antibacterial and antifungal activities showed that introducing a triazolium moiety in **223** and **224** would improve antibacterial and antifungal activities [87] (Scheme 61).

**Scheme 62:**
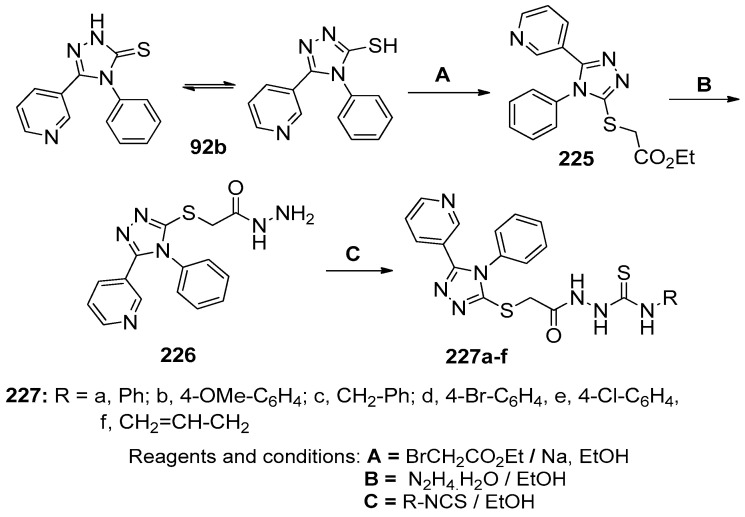
Synthesis of 1,2,4-triazole-3-yl)thio)acetyl)hydrazinecarbothioamides **227a**–**f**.

Reaction of 4-phenyl-5-(pyridin-3-yl)-1,2,4-triazole-3-thiol (**92b**) with ethyl bromoacetate gave 1,2,4-triazolthioacetate **225** which on reacting with hydrazine hydrate afforded the desired acetohydrazide **226**. Moreover, the synthesis of various *N*-substituted-2-(2-((4-phenyl-5-(pyridin-3-yl)-4*H*-1,2,4-triazole-3-yl)thio)acetyl)hydrazinecarbothioamides **227a**–**f** was achieved by reacting **226** with isothiocyanates (Scheme 62) [88]. 

**Scheme 63:**
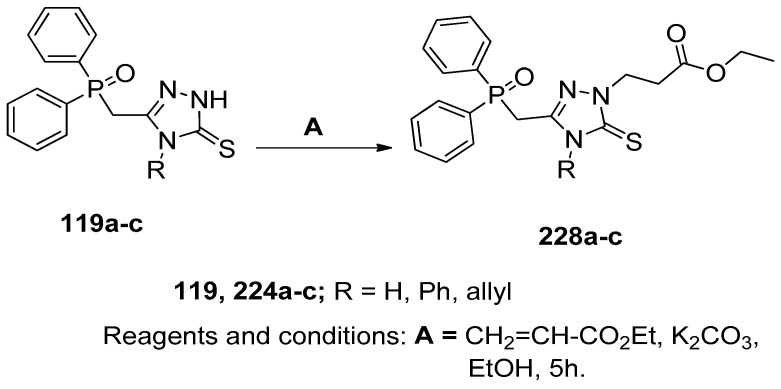
Synthesis of triazolethiones **228a**–**c**.

Subjecting 4-substituted 3-((diphenylphosphoryl)methyl)-1*H*-1,2,4-triazole-5(4*H*)-thiones **119a**,**b** with ethyl acrylate gave ethyl 3-(3-((diphenylphosphoryl)methyl)-4-substituted-5-thioxo-4,5-dihydro-1*H*-1,2,4-triazol-1-yl)propanoates **228a**–**c** [89] (Scheme 63).

**Scheme 64:**
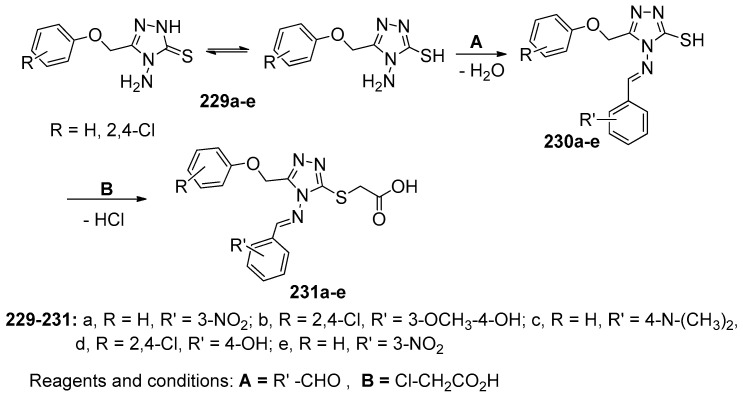
Synthesis of 1,2,4-triazole-3-yl)thio)acetic acid derivatives **231a**–**e**.

The Schiff bases of 4-amino-3-((phenoxy)methyl)-1*H*-1,2,4-triazole-5(4*H*)-thiones **230a**–**e** were synthesized upon reacting 4-aminotriazolethiones **229a**–**e** with different aldehydes. Additionally, reaction of **230a**–**e** with chloroacetic acid and catalyzation by pyridine gave 2-((4-(substituted benzylideneamino)phenoxymethyl-4*H*-1,2,4-triazole-3-yl)thio)acetic acid derivatives **231a**–**e** in 66–70% yields. The former compounds were screened for antimicrobial activities showing that compounds **231b** and **231d** have good antifungal activities against *Aspergillus niger*, *Cryptococcus neoformans*, and *Aspergillus fumigatus* at MIC of 0.25 μg/mL compared to the standard drug fluconazole with MIC of 1 μg/mL (Scheme 64) [90].

**Scheme 65:**
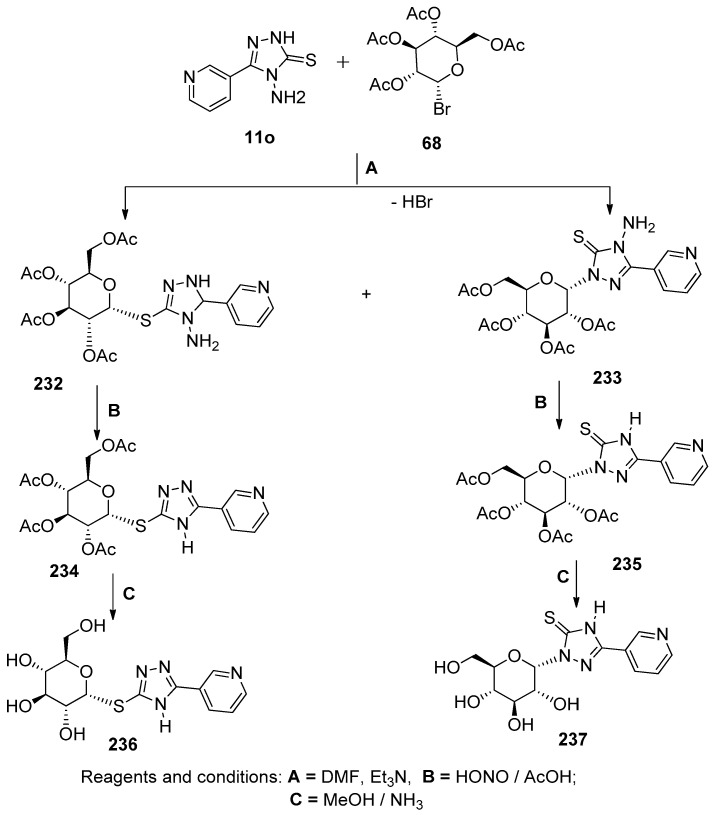
Synthesis of nucleosides **236** and **237**.

The reaction of **11o** with compound **68** afforded disubstituted aminotriazolethiones **232** in 57% and **233** in 40%. Deamination of compounds **232** and **233** was achieved using nitrous acid in acidic media to afford **234** and **235** in 75% and 85% yield, respectively. In addition, deacetylation of **234** and **235** with methanolic ammonia gave the free nucleosides **236** and **237** in 70% and 88% yield, respectively [91]. Screening of antibacterial and antifungal activities of these compounds revealed that *S*-alkylated derivatives **232**, **234**, and **236** have a higher inhibitory effect against *Aspergillus fumigatus*, *Syncephalastrum racemosum*, and *Staphylococcus aureus* as well as a lower inhibitory effect against *Penicillium italicum* and *Bacillus subtilis* compared to *N*-alkylated derivatives **233**, **235**, and **237** (Scheme 65) [91].

**Scheme 66:**
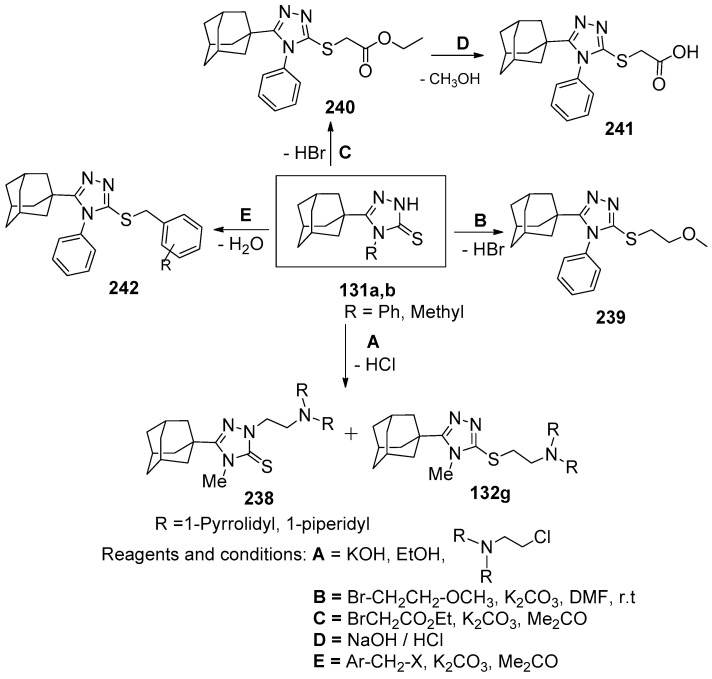
Synthesis of thio-4-phenyl-4*H*-1,2,4-triazoles **242a**–**e**.

Refluxing of 3-(adamantan-1-yl)-4-methyl-triazolethione **131b** with 2-aminochloride derivatives afforded *S*-(2-aminomethyl) and *N*-(2-aminomethyl) derivatives **238** and **132g** in 3:1 ratio, respectively [92]. Besides, 3-(adamantyl)-5-((2-methoxyethyl)thio)-4-phenyl-1,2,4-triazole **239** was obtained from the reaction of 1-bromo-2-methoxyethanone with 3-(adamantan-1-yl)-4-phenyl-1*H*-1,2,4-triazole-5(4*H*)-thione **131a**. However, in the case of ethyl bromo acetate, two products were formed, ethyl 2-((5-(adamantan-1-yl)-4-phenyl-1,2,4-triazole-3-yl)thio)acetate **240** that converted to 2-((5-(adamantan-1-yl)-4-phenyl-4*H*-1,2,4-triazole-3-yl)thio)acetic acid **241**. Moreover, reaction of aryl methyl halides with 3-(adamantan-1-yl)-4-phenyl-1*H*-1,2,4-triazole-5(4*H*)-thione **131a** afforded 3-(adamantan-1-yl)-5-((substituted benzyl)thio)-4-phenyl-4*H*-1,2,4-triazoles **242a**–**e** (Scheme 66). The synthesized compounds were tested against anti-inflammatory and antimicrobial activities. Compounds **240** and **241** exhibited good anti-inflammatory activity, whereas compounds **240**, **241**, and **242c**–**e** proved potent antibacterial activity against the tested microorganisms (Scheme 66) [92].

**Scheme 67:**
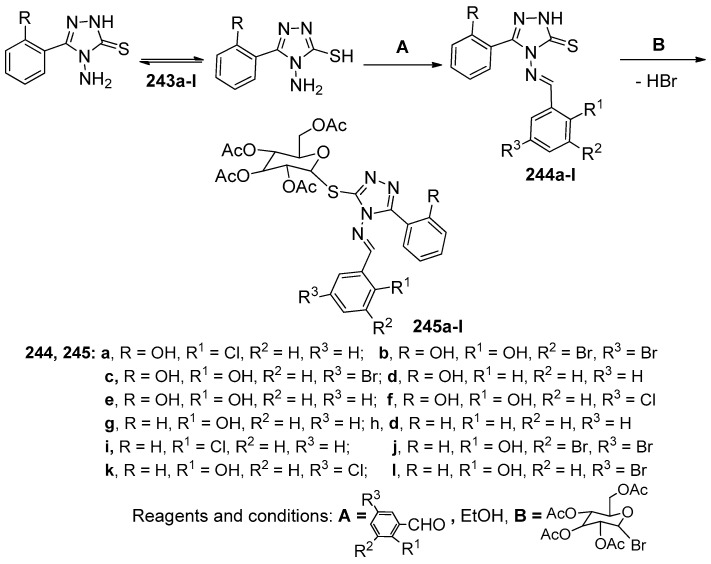
Synthesis of substituted triazoles **245a**–**i**.

The reaction of aminotriazolethiones **243a**–**i** with different aldehydes afforded various arylidenes **244a**–**l**, which upon reacting with **133** gave substituted triazoles **245a**–**i** (Scheme 67). The bioassay of antibacterial and antifungal activities of **245a**–**i** revealed that they have better antifungal than antibacterial activities; also, compounds **245b**,**c**,**f**,**j**,**k**,**l** showed excellent antifungal activity against *Candida albicans* with an MIC of 16 μg/mL [93]. 

**Scheme 68:**
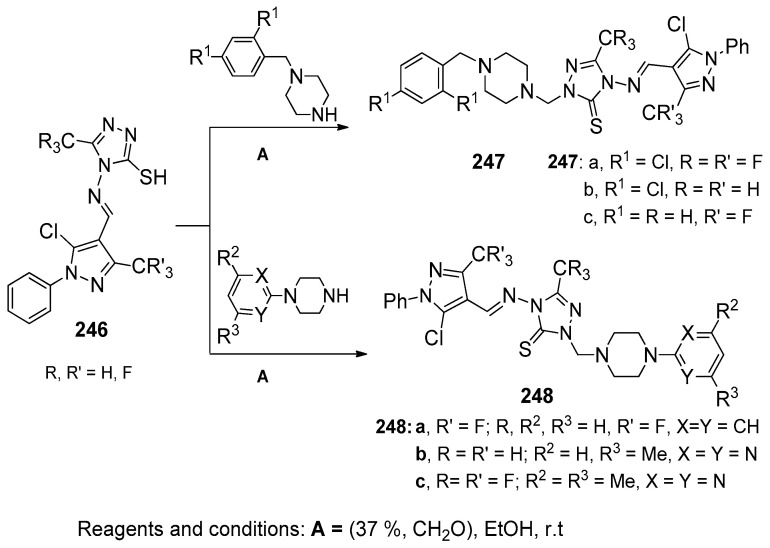
Synthesis of substituted triazoles **248a**–**c**.

Mannich reaction of arylidene derivatives of triazolethiols **246** with formaldehyde and benzyl piperazine or 4-substituted pyrimidyl/phenyl/pyridylpiperazine in ethanol at room temperature led to new Mannich bases **247a**–**c** and **248a**–**c** in 67–74% and 72–83% yields, respectively [94] (Scheme 68). The bioassay of the synthesized compounds revealed that these compounds could be used as new fungicides, whereas compounds **247a**–**c** exhibited higher and wider fungicidal activities comparable with that of control triadimefon (Scheme 68) [94].

**Scheme 69:**
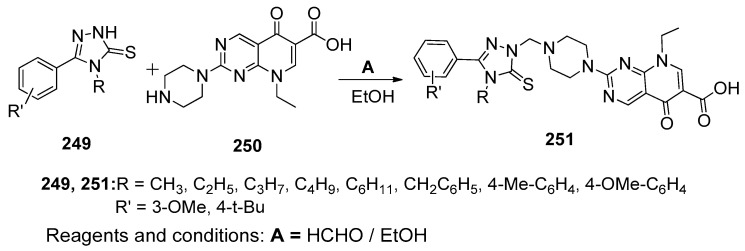
Reaction of triazolethione **249** with pipemidic acid **250**.

Reacting 4,5-disubstituted triazolethiones **249** with pipemidic acid **250** afforded 8-ethyl-2-(4-((3,4-disubstituted-5-thioxo-4,5-dihydro-1*H*-1,2,4-triazol-1-yl)methyl)piperazin-1-yl)-5-oxo-5,8-dihydropyrido[2,3-*d*]pyrimidine-6-carboxylic acid derivatives **251**, which exhibited significant antimicrobial activities [95] (Scheme 69).

**Scheme 70:**
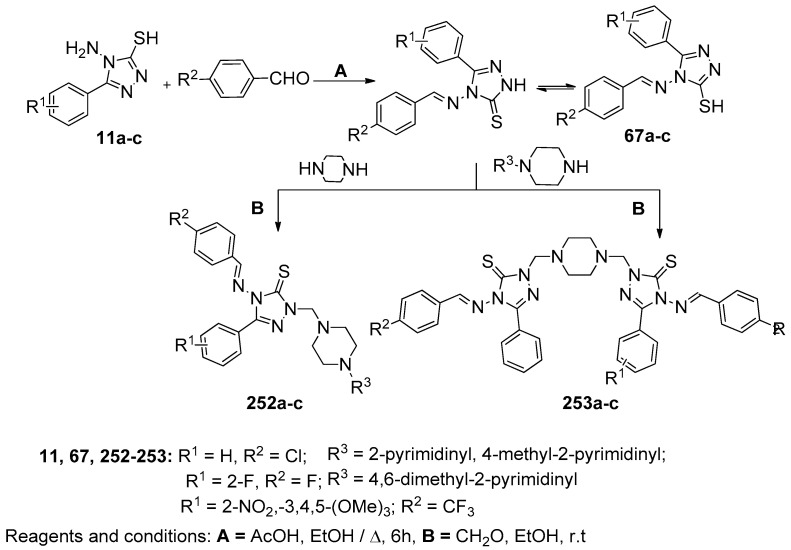
Synthesis of triazolethiones **252-** and **253a**–**c**.

Schiff bases of triazolethiones **67a**–**c** were obtained from the reaction of 4-substituted benzaldehyde with 4-amino-3-substituted-1,2,4-triazole-5-thiones **11a**–**c**. Besides, the reaction of compounds **67a**–**c**, formaldehyde, and 4-substituted piperazine gave the corresponding Mannich base **252a**–**c**. In addition, bis-Mannich base derivatives **253a**–**c** were synthesized in case of reaction of **67a**–**c** with piperazine (Scheme 70) [52].

**Scheme 71:**
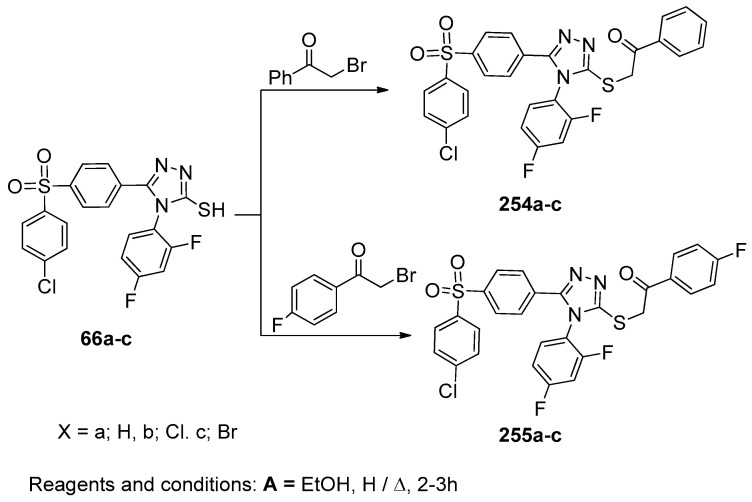
Synthesis *S*-alkylated triazoles **254a**–**c** and **255a**–**c**.

Reaction of 3,4-disubstituted triazolethioles **66a**–**c** with 2-bromoacetophenones gave ((3,4-disubstituted-1,2,4-triazole-3-yl)thio)-1-phenyl-ethanones **254a**–**c** in 70–82% yields and ((3,4-disubstituted-1,2,4-triazole-3-yl)thio)-1-(4-fluorophenyl)ethanones **255a**–**c** in 72–85% yields [96]. The screening of the antioxidant activity using the 2,2-diphenyl-1-picrylhydrazyl (DPPH) method revealed that the corresponding hydrazinecarbothioamides showed excellent antioxidant activity, while 1,2,4-triazole-3-thiones showed good antioxidant activity (Scheme 71) [96]. 

**Scheme 72:**
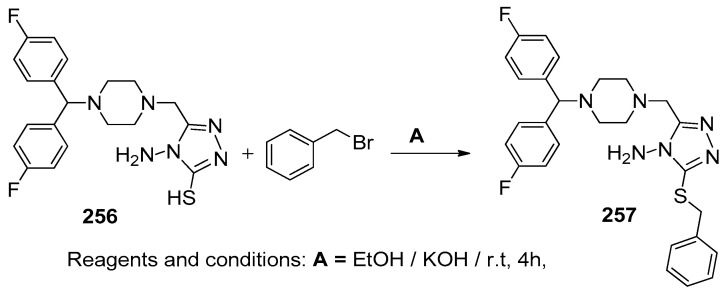
Synthesis of triazolebenzylthiol **257**.

The reaction of 4-amino-5-((4-(bis(4-fluorophenyl)methyl)piperazin-1-yl)methyl)-4*H*-1,2,4-triazole-3-thiol (**256**) with benzyl bromide gave 3-(benzylthio)-5-((4-(bis(4-fluorophenyl)-methyl)piperazin-1-yl)methyl)-4*H*-1,2,4-triazole-4-amine (**257**) in 72% yield (Scheme 72) [97]. 

### 3.2. Synthesis of Substituted Triazolethiones

#### Synthesis of Pyrazolo-1,2,4-triazoles

**Scheme 73:**
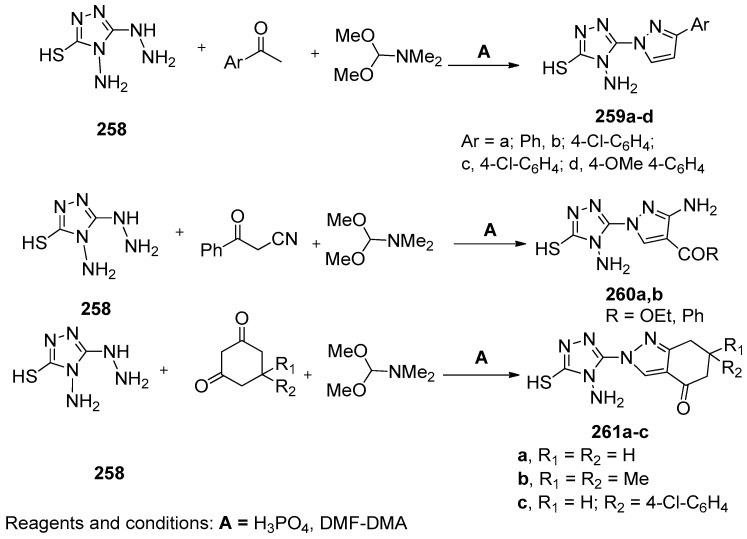
Synthesis of triazolethiols **259**–**261a**–**c**.

**Scheme 74:**
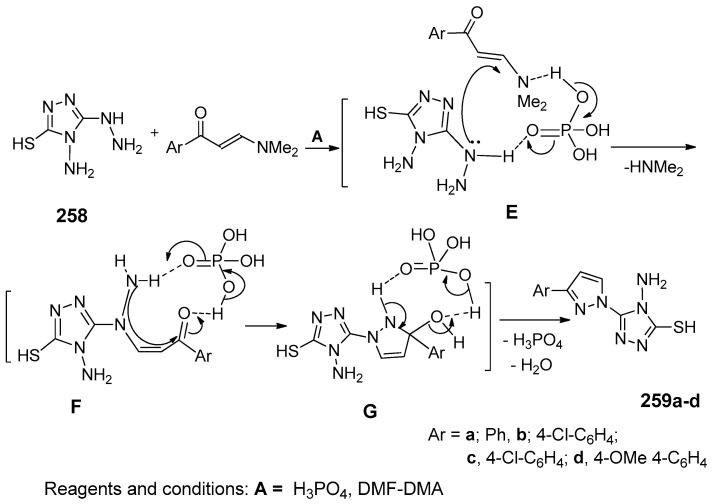
Mechanism describes formation of triazolethiols **259a**–**c**.

Compounds 4-Amino-5-(3-substituted pyrazolyl)triazolethiols **259a**–**d**, **260a**,**b**, and **261a**–**c** in 61–78% yields were synthesized *via* reacting 4-aminotriazole-3-thiol (**258**), dimethoxy-*N*,*N*-dimethylmethanamine, and carbonyl compounds using acidic media (orthophosphoric acid) as catalyst [98] (Scheme 73). The mechanism describing the role of orthophosphoric acid is presented in Scheme 74.

**Scheme 75:**
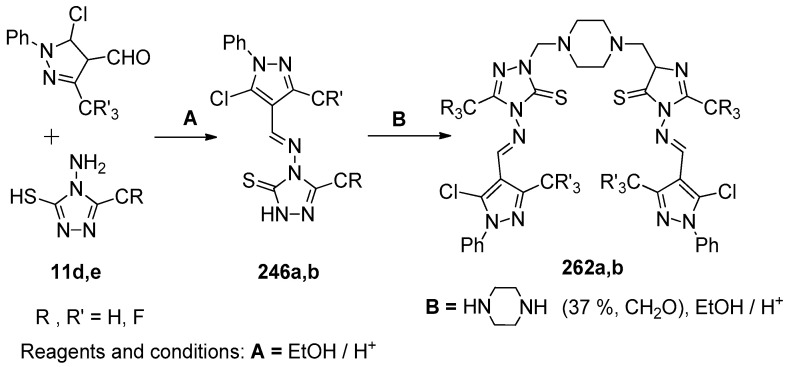
Synthesis of triazolethiones **262a**,**b**.

Condensation of 5-chloro-1-phenyl-3-(substituted)-1*H*-pyrazole-4-carbaldehyde with amino-triazolethiones **11d**,**e** furnished the corresponding Schiff bases **246a**,**b**. Bis-aminotriazolethiones **262a**,**b** can be obtained effectively in high yields (83–89% yields upon reaction of **246a**,**b** with piperazine and formaldehyde in ethanol at room temperature) (Scheme 75) [61]. 

### 3.3. Synthesis of Fused Triazoles

#### 3.3.1. Synthesis of Fused Pyrazolotriazoles

**Scheme 76:**
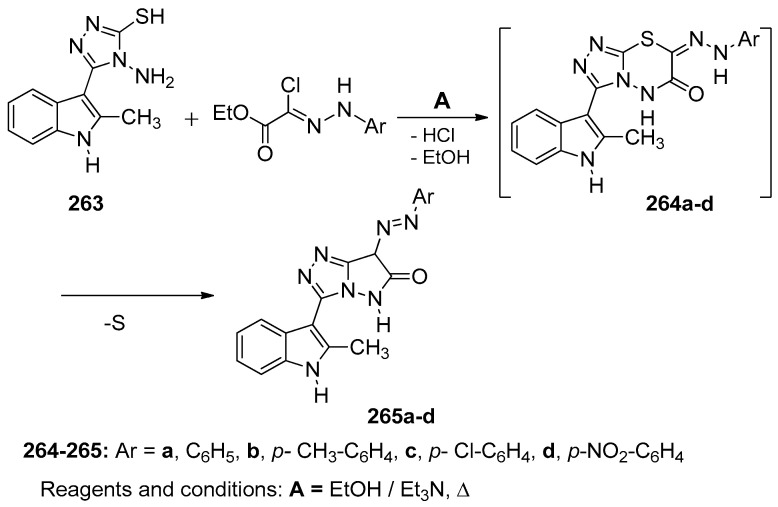
Synthesis of pyrazolotriazoles **265a**–**d**.

The synthesis of pyrazolotriazoles **265a**–**d** was easily established in 75–80% yields from the reaction of 4-amino-5-(substituted indole)-1,2,4-triazole-3-thiols **263a**–**d** with *N*-arylhydrazonoacetates using basic media of triethylamine *via* the intermediate **264a**–**d**. Desulfurization and ring contraction of compounds **264a**–**d** gave the target compound **265a**–**d** (Scheme 76) [99].

#### 3.3.2. Synthesis of Thiazolotriazoles

**Scheme 77:**
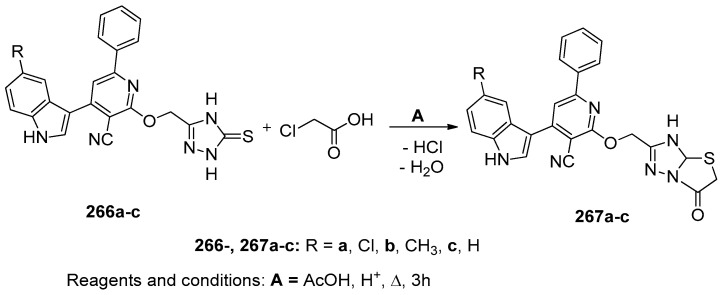
Synthesis of thiazolotriazoles **267a**–**c**.

The condensation reaction of 3-substituted-1,2,4-triazole **266a**–**c** with chloroacetic acid in acidic media afforded thiazolotriazoles **267a**–**c** in 65–69% yields. Interestingly, the synthesized compounds were screened for their antioxidant and antimicrobial activities [100] (Scheme 77). Compound **267a** exhibited effective antimicrobial activity towards all the tested organisms.

**Scheme 78:**
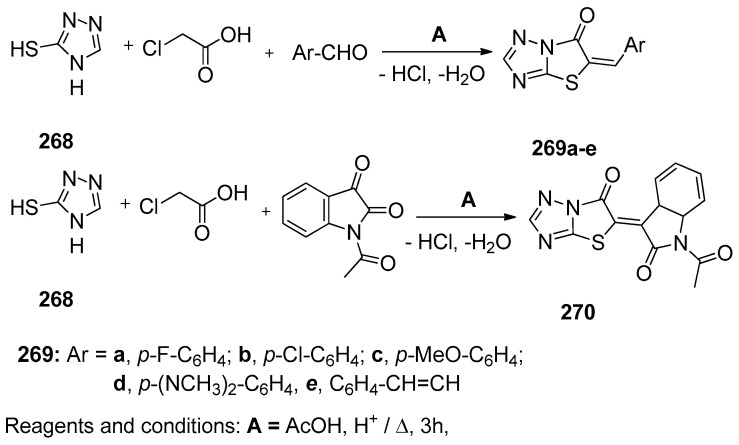
Synthesis of triazolo[1,3,4]-thiadiazoles **269a**–**e** and **270**.

However, in case of refluxing of 4*H*-1,2,4-triazole-3-thiol (**268**) with chloroacetic acid and different aldehydes or isatin derivatives in acidic media, various 5-substituted arylidene-thiazolotriazoles **269a**–**e** in 49–68% yields or (*Z*)-5-(1-acetyl-2-oxo-1*H*-indol-3(2*H*,3*aH*,7*aH*)-ylidene)thiazolo[3,2-*b*]-1,2,4-triazol-6(5*H*)-ones **270** were efficiently synthesized in 40% yield (Scheme 78) [101]. Screening of the obtained compounds for anticancer activity revealed that 5-arylidene-[1,3]thiazolo[3,2-*b*][1,2,4]triazol-6-ones **269a**–**e** exhibited more potent anticancer activity than respective amides [101].

**Scheme 79:**
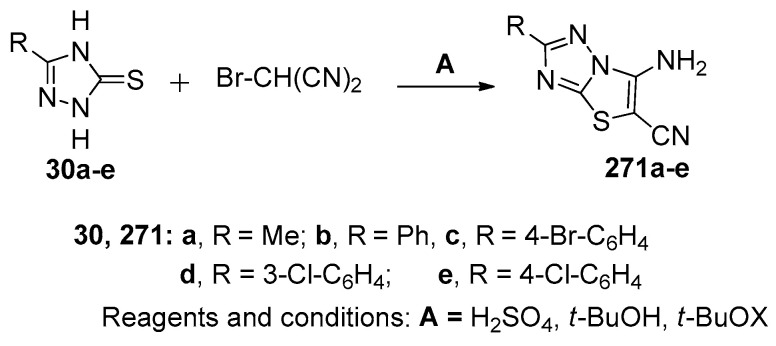
Synthesis of thiazolo-1,2,4-triazole carbonitriles **271a**–**e**.

The reaction of different bromomalononitrile derivatives with 3-substituted triazolethiones **30a**–**e** produced the corresponding thiazolo-1,2,4-triazole carbonitriles **271a**–**e** [102] (Scheme 79).

**Scheme 80:**
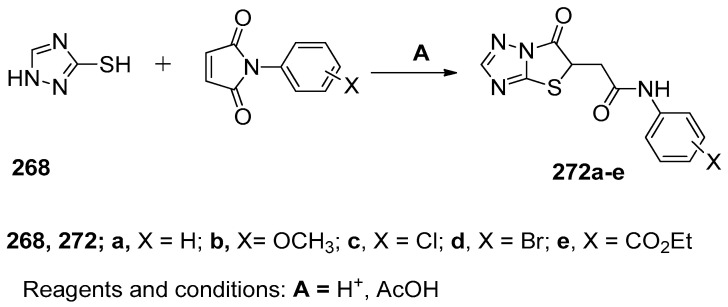
Synthesis of dihydrothiazolo[3,2-*b*][1,2,4]triazol-5-yl)acetamides **272a**–**e**.

The synthesis of *N*-aryl-2-(6-oxo-5,6-dihydrothiazolo[3,2-*b*][1,2,4]triazol-5-yl)acetamides **272** was achieved in 60–87% yields from the reaction of **268** with *N*-arylmaleimides in acidic media. It was established from the structure–activity relationship of these compounds that halo-substituted derivatives have a considerable increase in anticancer activities [103] (Scheme 80).

**Scheme 81:**
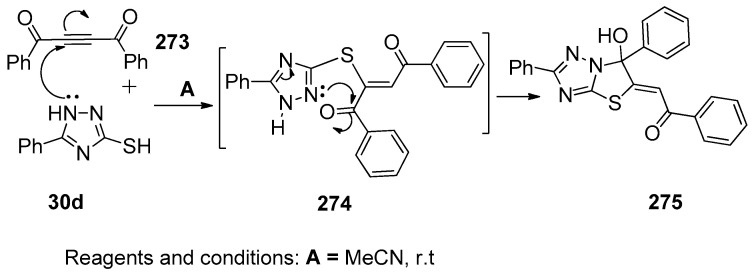
Synthesis of thiazolotriazole **275**.

Shah et al. [104]. have reported the synthesis of thiazolotriazoles **275** in 77–85% yields via refluxing of 5-phenyl-1,2,4-triazole-3-thiol (**30d**) with dibenzoylacetylene (**273**) through the intermediate **270** (Scheme 81).

#### 3.3.3. Synthesis of Triazolothiadiazoles

**Scheme 82:**
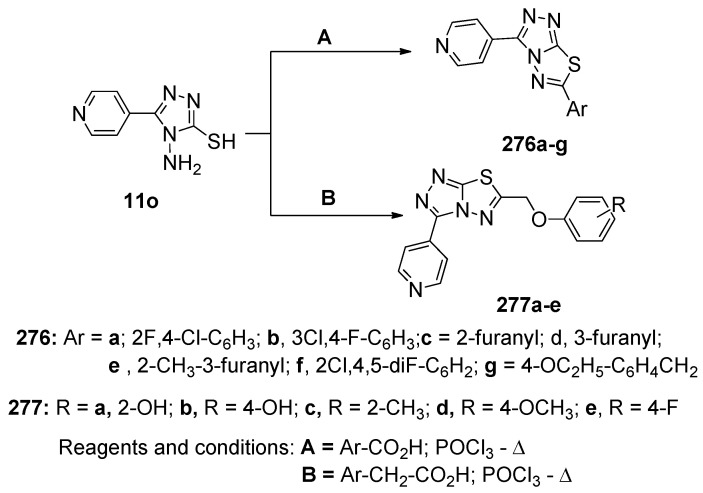
Synthesis of triazolo[1,3,4]-thiadiazoles **276** and **277**.

Triazolo[3,4-*b*][1,3,4]thiadiazoles **276a**–**g** and **277a**–**e** were obtained from refluxing of different aromatic carboxylic acids with **11o** in the presence of phosphorous oxychloride [105]. Screening of the synthesized compounds against *lung carcinoma* (H157) and *kidney fibroblast* cell lines (BHK-21) showed that compound **277d** has the highest inhibition activity of 74.0% for BHK-21 cells which is the same as that of standard drug vincristine (74.5%). Compound **276c**,**d**,**g** showed less inhibition values, and triazolothidiazole **276a** was the most potent compound with inhibition value of 85.5%, whereas compounds **276b**,**f** and **277a** exhibited less inhibition values (Scheme 82) [105]. 

**Scheme 83:**
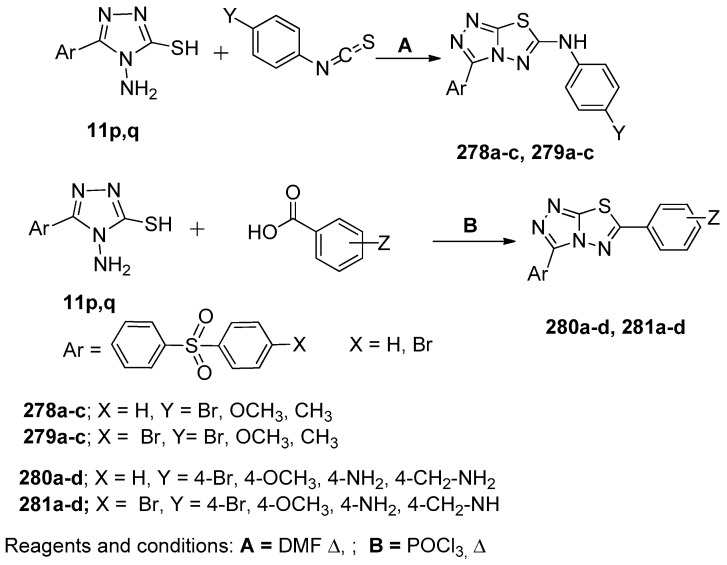
Synthesis of triazolo[1,3,4]-thiadiazoles **278**–**281a**–**c**.

The synthesis of fused heterocyclic[1,2,4]triazolo[3,4-*b*]-1,3,4-thiadiazoles **278a**–**c** and **281a**–**c** was done by the reaction of 4-amino-5-(4-((4-X-phenyl)sulfonyl)phenyl)-4*H*-1,2,4-triazole-3-thiol (**11p**,**q**) with aryl isothiocyanates or with various aromatic acids. Antimicrobial screening of the synthesized compounds showed that they had good antimicrobial activity (Scheme 83) [106].

**Scheme 84:**
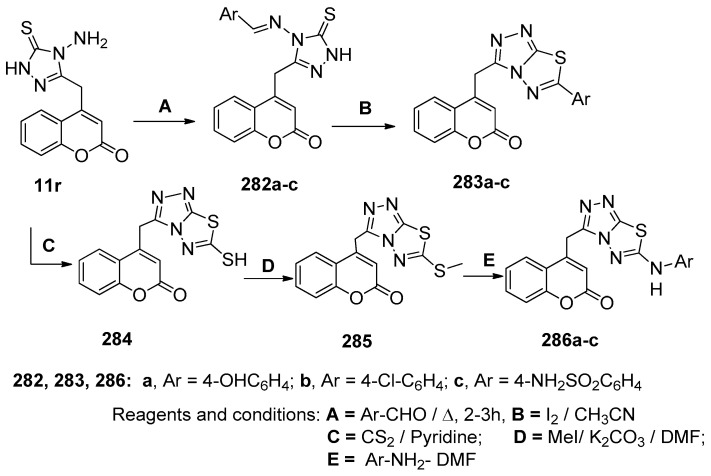
Synthesis of chromone derived by triazolethiadiazines **283a**–**c** and triazolo[3,4-*b*][1,3,4]thiadiazoles **286a**–**c**.

When 3-substituted methylaminotriazolethione **11r** was condensed with different aldehydes, arylidene derivatives of triazolethiones **282a**–**c** were obtained in 54–66% yield, whereas triazolothiadiazoles **283a**–**c**, in 48–74% yields, were synthesized from the reaction of compound **282a**–**c** with iodine. In addition, 6-mercapto-1,2,4-triazolothiadiazoles **284** were synthesized upon reaction of **11r** with CS_2_ in pyridine. The synthesis of 4-((6-(ethylthio)-[1,2,4]triazolo[3,4-*b*][1,3,4]-thiadiazol-3-yl)methyl)-2*H*-chromen-2-one **285** was occurred from reaction of **11r** with methyl iodide in basic media in 55–75% yields. Moreover, 6-methylthio derivative **285** reacted with different aromatic amines to give triazolothiadiazoles **286a**–**c** (in 55–75% yields). The obtained compounds were evaluated in vitro as anticancer agents in the human colon cancer (HCT 116) cell line where the aminosulfanyl derivative **286c** exhibited high anticancer activity (Scheme 84) [37]. 

#### 3.3.4. Synthesis of 1,2,4-Triazolothiazines

**Scheme 85:**
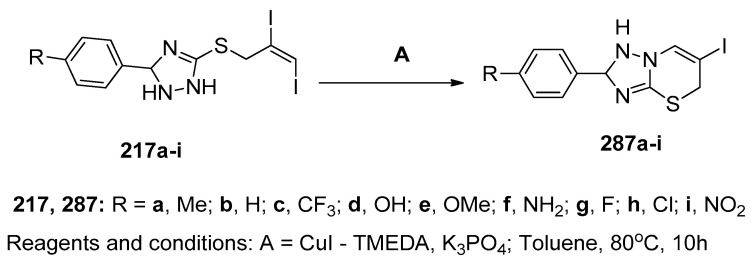
Synthesis of triazolothiadiazines **287a**–**i**.

The synthesis of triazolothiazines **287a**–**i** (in 48–72% yields) was done from the reaction of compound **217a**–**i** with CuI and tetramethylethylenediamine (TMEDA) using basic media (Scheme 85) [107].

**Scheme 86:**
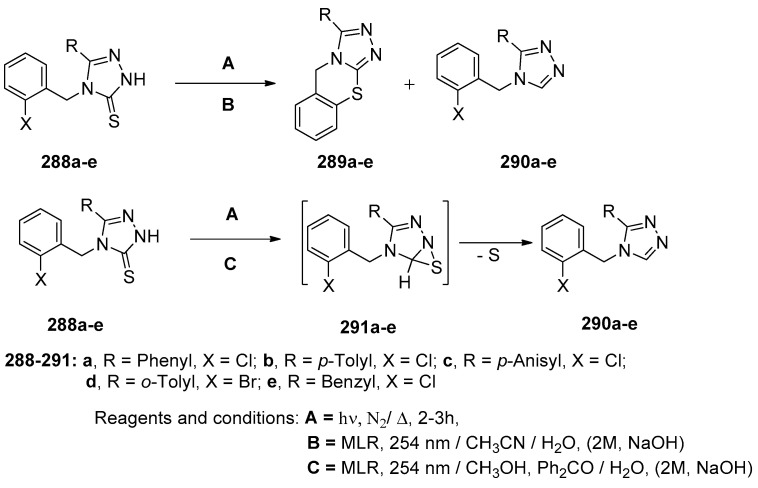
Synthesis of triazoles **290a**–**e**.

UV irradiation of disubstituted triazole-5(4*H*)-thione **288a**–**e** under basic conditions gave a mixture of 3-substituted triazolothiazines **289a**–**e** and 3,4-disubstituted-1,2,4-triazoles **290a**–**e** according to the concentration of the base used. However, irradiation of **288a**–**e** in presence of acetophenone and only compounds **290a**–**e** was observed [108,109] (Scheme 86).

**Scheme 87:**
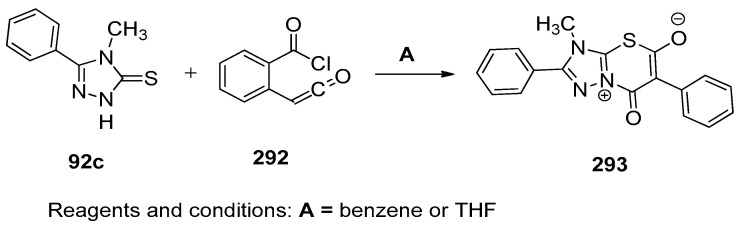
Synthesis of triazolothiadiazines **293**.

Reaction of **92c** with 2-chlorobenzoylketene **292** afforded 3-methyl-7-oxo-2,6-diphenyl-3,7-dihydro-[1,2,4]triazolo-[5,1-*b*][1,3]thiazin-8-ium-5-olate **293** in 66% yield [110] (Scheme 87).

#### 3.3.5. Synthesis of 1,2,4-Triazolothiadiazines

**Scheme 88:**
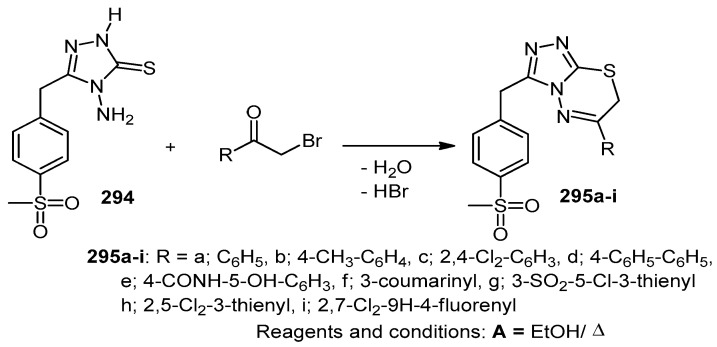
Synthesis of triazolothiadiazines **295a**–**j**.

Cyclocondensation of 4-amino-3-(4-(methylsulfonyl)benzyl)-1*H*-1,2,4-triazole-5(4*H*)-thione (**294**) with different substituted phenacyl bromide derivatives in ethanol afforded 6-substituted-3-(4-(methylsulfonyl)benzyl)-7*H*-[1,2,4]triazolo[3,4-*b*][1,3,4]-thiadiazines **295a**–**i** (Scheme 88). The antimicrobial activity of the synthesized compounds showed that triazolothiadiazines **295b**,**e**,**f**,**h** have significant antibacterial and antifungal activities against all the tested microorganisms [111].

**Scheme 89:**
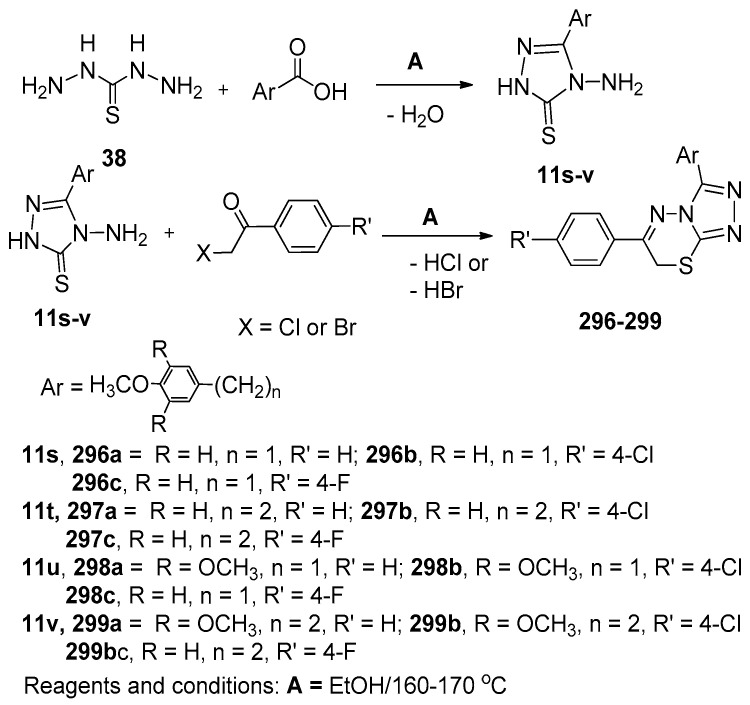
Synthesis of triazolothiadiazines **296**–**299**.

Refluxing of different carboxylic acids with thiocarbohydrazides **38** led to 4-amino-3-substituted-1,2,4-triazole-5-thiones **11s**–**v**. Treatment of **11s**–**v** with phenacyl bromide or chloride derivatives yielded triazolothiadiazines **296**–**299** [112] (Scheme 89). Interestingly, compounds **297b**, **299b**, and **299c**, having either a chloride or fluoro substituent on the phenyl ring, gave better analgesic and anti-inflammatory activities and less ulcerogenic risk, along with minimum lipid peroxidation [112].

**Scheme 90:**
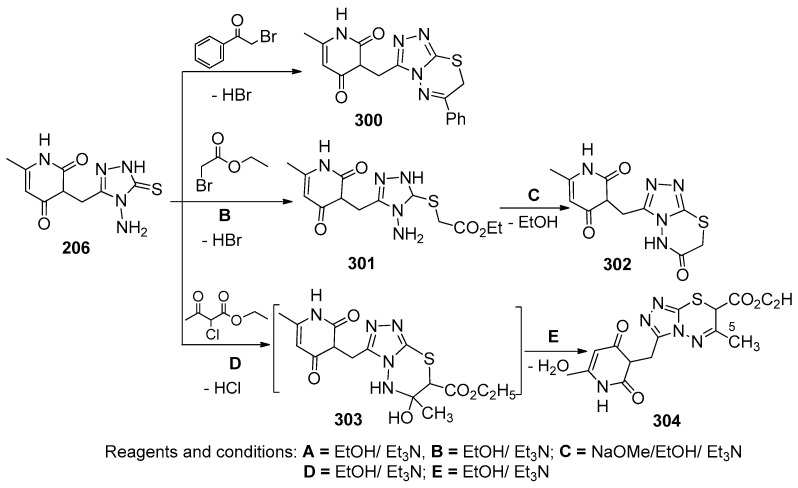
Synthesis of triazolothiadiazine **304**.

Various electrophilic reagents reacted with **206** to give different triazolothiadiazines **300**, **302**, and **304**. Ethyl 2-((4-amino-5-((4-methyl-2,6-dioxo-2,3-dihydropyrimidin-1(6*H*)-yl)methyl)-4*H*-1,2,4-triazole-3-yl)thio)acetate **301** was obtained from the reaction of **206** with ethyl bromoacetate which cyclized using sodium methoxide to give triazolothiadiazine **302**. However, reacting **206** with ethyl 2-bromoacetate and ethyl-2-chloroacetoacetate gave substituted triazolothiadiazine **300** and ethyl-substituted triazolo-[3,4-*b*][1,3,4]thiadiazine-7-carboxylate **304**, respectively (Scheme 90) [113]. 

**Scheme 91:**
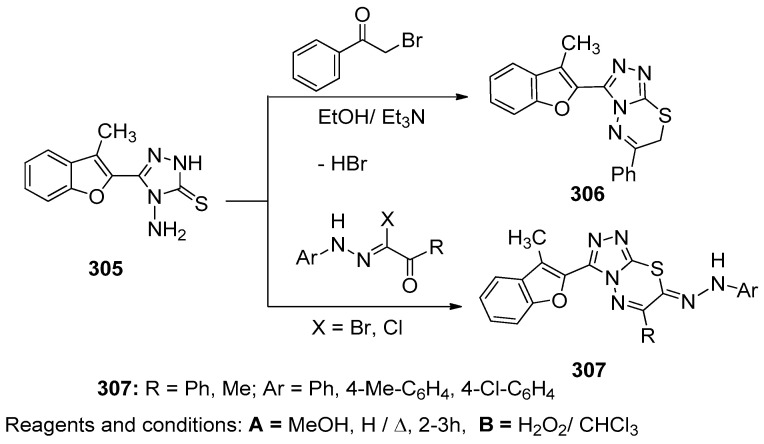
Synthesis of triazolothiadiazines **307**.

In a similar reaction, the reaction of (3-methylbenzofuran-2-yl)triazolethione **305** with either 2-bromoacetophenone or hydrazonyl halides produced the corresponding 3-(3-methylbenzofuran-2-yl)-triazolothiadiazine **306** and (2-arylhydrazono)triazolothiadiazine derivative **307**, respectively [114] (Scheme 91).

**Scheme 92:**
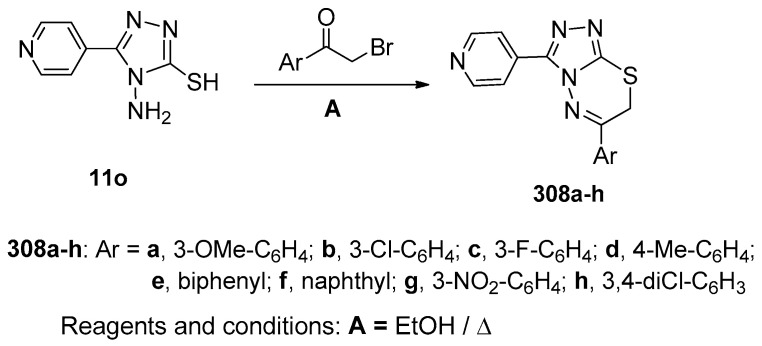
Synthesis of triazolothiadiazines **308a**–**h**.

It was reported that 1,2,4-triazole-3-thiol **11o** reacted with various bromoacetophenone to yield the corresponding 3,6-disubstituted triazolothiadiazine **308a**–**h**. The anticancer activity of these compounds was studied against H157 and BHK-21M cell lines showing that compound **308c** was a potent inhibitor of H157 cells having 78.6% inhibition and compounds **308a** and **308d** were potent inhibitors in cancer therapy against BHK-21 cells with 73.3% and 72.6% inhibition, respectively [115] (Scheme 92).

**Scheme 93:**
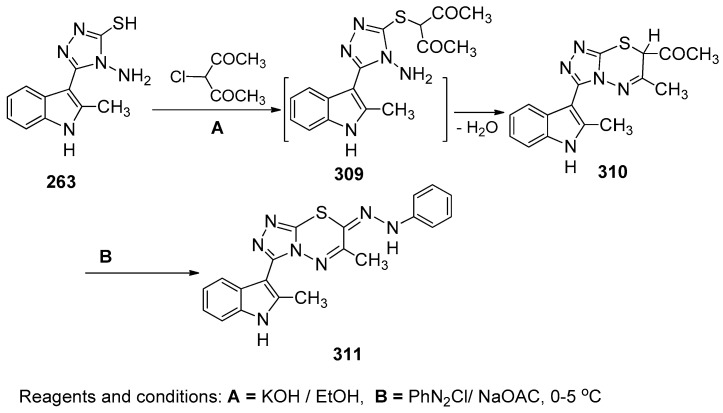
Synthesis of triazolothiadiazine 311.

4-Amino-5-(2-methyl-1*H*-indol-3-yl)-4*H*-1,2,4-triazole-3-thiol (**263**) was successfully cyclized to give triazolo[3,4-*b*][1,3,4]thiadiazine (**310**) *via* reacting with 3-chloropentane-2,4-dione through the intermediate **309**. Furthermore, diazotization occurred to compound **310** and chlorophenyldiazene to give **311** (Scheme 93) [116].

**Scheme 94:**
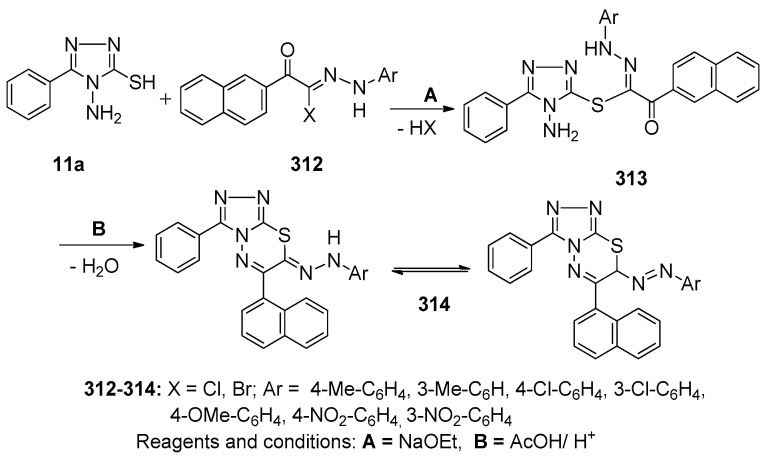
Synthesis of triazolothiadiazines **314**.

On reaction of **11a** with *N*′-arylacetohydrazonoyl halides **312** in the presence of sodium ethoxide, the reaction gave the corresponding 1,2,4-triazole-3-yl-2-(naphthalen-2-yl)-*N*′-arylethanehydrazonothioates **313**. Cyclization of **313** in acidic media gave the target compound triazolothiadiazine **314** [117] (Scheme 94).

**Scheme 95:**
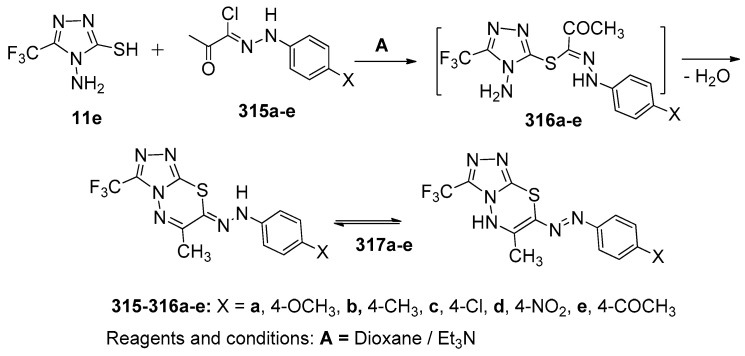
Synthesis of [1,2,4]triazolo[3,4-*b*]-[1,3,4]thiadiazines **317a**–**e**.

Refluxing of **11e** with *N*-aryl-2-oxopropanehydrazonoylchloride **315a**–**e** afforded (*Z*)-6-methyl-7-(2-aryllhydrazono)-3-(trifluoromethyl)-7*H*-[1,2,4]triazolo[3,4-*b*]-[1,3,4]thiadiazine (**317**) *via* the formation of intermediate **316** [118]. Screening of the anticancer activities revealed that compounds **316a**,**e** were the most active inhibitors against HEPG-2 cell line, whereas compound **316a** was active against HCT cell line [118] (Scheme 95).

## 4. Conclusions

In this review, we are trying to focus attention on the routes of triazole-thione synthesis. Since, triazolethione-thiols have gained considerable importance in medicinal chemistry, due to their broad spectrum as antiviral, antibacterial, anticancer, etc. agents, their synthesis has become of great interest. We also give spots on the biology of the target molecule as prospective antiviral drugs.
